# Proximity-based proteomics (BioID) uncovers the Rho GTPase interactome in kidney podocytes

**DOI:** 10.3389/fcell.2025.1625950

**Published:** 2025-11-11

**Authors:** Sajida Ibrahim, Jun Matsuda, Zachary W. Nurcombe, Jonathan Boulais, Lamine Aoudjit, Emily Foxman, Cyril Kazan, Soichiro Suzuki, Simon Leclerc, Naoyuki Shimada, Thomas Kitzler, Jean-François Coté, Tomoko Takano

**Affiliations:** 1 Research institute of the McGill University Health Centre (RI-MUHC), Montreal, QC, Canada; 2 Department of Nephrology, Graduate School of Medicine, The University of Osaka, Suita/Osaka, Japan; 3 Montreal Clinical Research Institute (IRCM), Montreal, QC, Canada

**Keywords:** BioID, Cdc42, Rac1, RhoA, KIAA1522, ARHGEF12, podocyte

## Abstract

**Introduction:**

Podocyte injury causes proteinuria. Rho GTPases play critical roles in regulating the podocyte cytoskeleton, and their alteration leads to foot process effacement. Yet, their signaling networks remain poorly understood.

**Methodology:**

To address this, we mapped the interactomes of RhoA, Rac1, and Cdc42 in human podocytes using proximity-dependent biotin identification (BioID) labeling.

**Results and discussion:**

Our BioID analysis detected a total of 1927 interactions with AvgP ≥ 0.95. Approximately 50% of the interactions are unique to podocytes compared to interactions in HEK293 and HeLa cells, with enrichment in pathways related to cell adhesion and shape organization. *KIAA1522* emerged as a Rac1/Cdc42 interactor. *KIAA1522* knockout reduced cellular projection formation in podocytes, while *KIAA1522* knockdown in zebrafish resulted in foot process effacement. Additionally, we identified 20 guanine nucleotide exchange factors (GEFs), with 11, 8, and 5 interacting with RhoA, Rac1, and Cdc42, respectively. Analysis of public scRNA-seq datasets identified RhoA regulators as highly enriched in podocytes. Knockout of most RhoA GEFs reduced RhoA activity, with ARHGEF12 having the greatest effect. Our study defined key upstream regulators and downstream effectors of Rho GTPases in podocytes, identifying *KIAA1522* as a novel Cdc42 effector and ARHGEF12 as a key RhoA regulator.

## 1 Introduction

Podocytes are highly specialized epithelial cells that are critical for plasma filtration in the kidney glomerulus. They possess interdigitated actin-based projections, known as foot processes, which wrap around capillaries and form filtration structures called the slit diaphragms. Podocyte injury and the subsequent disruption of the glomerular filtration barrier result in proteinuric kidney diseases, which could eventually lead to kidney failure (Kriz and Lemley, 2015). Deciphering the molecular mechanisms underlying cytoskeleton remodeling is crucial to understanding podocyte injury and developing targeted therapies. Previous studies, including ours, have shown that the Rho family of small GTPases (Rho GTPases) plays critical roles in regulating the podocyte cytoskeleton. The dysregulation of Rho GTPase activity results in foot process effacement, podocyte detachment, and subsequent proteinuria (Blattner et al., 2013; Robins et al., 2017; Zhu et al., 2011; Zhu et al., 2013). The current study aims to fill an important knowledge gap regarding the regulation of Rho GTPase activities in podocytes.

Rho GTPases are molecular switches that transduce upstream signals to downstream effectors by alternating between an inactive GDP-bound state and an active GTP-bound state. This is achieved through the interaction with various upstream regulators, including 1) guanine nucleotide exchange factors (GEFs) that activate Rho GTPases by promoting the exchange of GDP for GTP and 2) GTPase-activating proteins (GAPs) that deactivate Rho GTPases by promoting GTP hydrolysis into GDP (Mouawad et al., 2013). Rho GTPases can modulate a large spectrum of cellular processes ranging from transcriptional regulation to membrane trafficking, cell-adhesion, morphogenesis, and actin/dynamic cytoskeleton remodeling. This is mediated by unique signaling cascades that are regulated by specific GEFs and GAPs (Matsuda et al., 2021).

The Rho family of small GTPases is represented by the three prototypical proteins—RhoA, Rac1, and Cdc42—but the entire family consists of 21 small G-proteins, most of which remain poorly characterized. In addition, approximately 82 GEFs (Fort and Blangy, 2017; Laurin and Cote, 2014) and 69 GAPs (Amin et al., 2016; Tcherkezian and Lamarche-Vane, 2007) have been identified in humans, but their interplay with Rho GTPases is complex and remains poorly understood in general and, in particular, in podocytes. This is due, in part, to the sparsity of podocytes within the kidney and their under-representation in bulk expression analyses.

Proximity-based biotinylation assay (BioID) is an efficient technique that captures transient protein–protein interactions in a near-physiological context and identifies interactors across various cellular compartments, offering advantages over traditional protein interaction techniques (Kim and Roux, 2016; Roux et al., 2018). To gain a deeper understanding of Rho GTPase signaling in podocytes, we used proximity-based proteomics (BioID) and publicly available single-cell RNA-sequencing (scRNA-seq) datasets of whole kidneys or kidney glomeruli and performed a comprehensive analysis of Rho GTPases and their interactors, including GEFs and GAPs. Our results, for the first time, demonstrate the Rho GTPase interactome in human podocytes, where the RhoA pathway is dominant. Furthermore, we characterized the function of a novel Rho GTPase effector KIAA1522 and all RhoA-targeting GEFs in podocytes.

## 2 Materials and methods

### 2.1 Cell culture

Immortalized human podocytes were obtained from Dr. Moin Saleem (Saleem et al., 2002). Podocytes were cultured in RPMI (Wisent Inc., 350–000-CL) supplemented with 10% FBS and 1% penicillin/streptomycin (PS) and were maintained under permissive conditions (33 °C) with 5% CO_2_. HEK293 cells were maintained in DMEM containing 10% FBS and 1% PS at 37 °C with 5% CO_2_.

### 2.2 Proximity-dependent biotin identification assay

Proximity-dependent biotin identification (BioID) bait constructs were gifts from Drs. Anne-Claude Gingras (Lunenfeld-Tanenbaum Research Institute) and Jean-Francois Coté (Montreal Clinical Research Institute). Wild-type Rho GTPases (RhoA WT, Rac1 WT, and Cdc42 WT), nucleotide-free (NF) mutants (RhoAG17A, Rac1G15A, and Cdc42G15A), and constitutively active mutants (RhoAG14V, Rac1G12V, and Cdc42G12V) were subcloned into pSTV6 (gift from Dr. Anne-Claude Gingras), a tetracycline-inducible lentiviral vector that contains BirA with puromycin N-acetyltransferase (PAC) as a reporter. Empty MYC-BirA vector and GFP subcloned into pSTV2-N-BirA*-Flag were used as negative controls (Samavarchi-Tehrani et al., 2018).

#### 2.2.1 Transduction

Human podocytes were transduced using fresh lentiviral particles produced in HEK293 cells. Virus-containing supernatants were added to human podocytes for 24 h. Puromycin (Wisent Inc.) was added 48 h later to select puromycin-resistant transduced cells, followed by polyclonal expansion for further experiments. Bait protein expression in podocytes across three independent sample sets was validated by Western blot (Supplementary Figure S7).

#### 2.2.2 Protein extraction and mass spectrometry

The BioID experiment was performed as described previously (Roux et al., 2012). In brief, human podocytes were incubated for 16 h with 1 μg/mL doxycycline (Sigma, D9891) and 50 μM biotin (BioShop, BIO302). Only for constitutively active mutants, cells were cultured with 0.1 μg/mL doxycycline (Sigma, D9891) and tetracycline-free FBS to avoid cytotoxicity. Cells were collected by scraping in phosphate-buffered saline (PBS) and were pelleted at 800 rpm for 5 min at 4 °C, followed by snap-freezing on dry ice. To extract proteins, the pellets were incubated for 1 h at 4 °C with RIPA buffer [50 mM Tris (pH 7.4), 150 mM NaCl, 1% NP40, 0.5% sodium deoxycholate, 0.1% SDS, and 1 mM EDTA] supplemented with 1 mM PMSF and protease inhibitor cocktail (cOmplete™, Roche 11836170001, Indianapolis, IN, USA). Lysates were then sonicated and centrifuged. Biotinylated proteins in the supernatant were pulled down using Dynabeads MyOne Streptavidin C1 (Thermo Fisher Scientific, Durham, NC, USA) for 3 h at 4 °C. Finally, the beads were rinsed five times in RIPA, followed by four washes in low detergent buffer [25 mM Tris (pH7.4), 100 mM NaCl, and 0.025% SDS]. Samples were then analyzed by mass spectrometry.

#### 2.2.3 Statistical analysis

Statistical analysis was performed as described previously (Bagci et al., 2020; Nahle et al., 2022). In brief, Significance Analysis of INTeractome (SAINTexpress) analyses were performed on the detected interactions. Proximity interactions displaying a SAINT average probability (AvgP) ≥ 0.95 (below the Bayesian 1% false-discovery rate (FDR) estimate) were retained and considered of high confidence.

### 2.3 Transcriptomic analysis of public datasets

He et al. (2021) dataset: RPKM data of scRNA-seq on human glomeruli from the He et al. (2021) dataset (GSE160048) were downloaded from the GEO database, and podocytes were identified based on the expression of *NPHS1* and *NPHS2* genes. Muto et al. (2021) dataset: for the single-nucleus RNA sequencing (snRNA-seq) data from healthy kidneys, normalized Seurat objects were downloaded from the GitHub repository. Diabetic kidney disease dataset: log2 expression data were extracted from Wilson et al. (2019). Human adult kidney dataset: the expression levels of BioID-identified GEFs were examined in various human kidney cell types using the human adult kidney data from Subramanian et al. (2019), available on the Single Cell Portal (https://singlecell.broadinstitute.org/single_cell).

### 2.4 Wound healing assay

Podocytes were cultured in a 96-well IncuCyte® ImageLock microplate coated with collagen at 33 °C and then serum-starved at 37 °C in RPMI containing 1% FBS for 2 h before wound induction. Confluent monolayers were scratched using the IncuCyte 96-well Wound Maker (Sartorius, Ann Arbor, MI, USA), and cells were kept at 37 °C up to 24 h. The podocyte migration rate was calculated using the percentage of wound confluence generated by IncuCyte Analysis Software (Sartorius, Ann Arbor, MI, USA). The percentage of the scrambled control was calculated based on the 6- to 8-hour time points.

### 2.5 Cellular projection formation assay

Podocytes were cultured in 12-well plates on day 0. On day 1, podocytes were treated with EGF (100 ng) and placed in a 37 °C incubator. Ten hours after EGF treatment, 16 snapshots of each well were taken using IncuCyte S3 (Essen BioScience, Ann Arbor, MI, USA). Cellular projections were counted manually and normalized to the percentage of cell confluence, as calculated by IncuCyte software.

### 2.6 Mouse glomeruli isolation

Mouse glomeruli were isolated through differential sieving, as described previously (Zhu et al., 2008), and lysed in IP buffer for 30 min on ice. Proteins were eluted in Laemmli buffer and then assayed by Western blot.

### 2.7 Co-immunoprecipitation

HEK293 cells were transfected with the KIAA1522-mCherry plasmid for 16 h and then lysed in IP buffer. Cell lysates were incubated with KIAA1522 antibody or rabbit IgG overnight at 4 °C. Protein A agarose beads (Santa Cruz, sc-2001, Dallas, TX, USA) were added for 1 h at 4 °C on rotation. Beads were washed three times, and proteins were eluted in Laemmli buffer and then assayed by Western blot.

### 2.8 GST pull-down

Cells were lysed in TNE buffer. Cell lysates were incubated with streptavidin beads fused to GST-empty or GST-IRSp53 for 1 h at 4 °C on rotation. Beads were washed three times, and proteins were eluted in Laemmli buffer and then assayed by Western blot.

### 2.9 Immunoblotting

Cells were lysed in IP buffer. Proteins were separated by SDS-PAGE and transferred to nitrocellulose membranes; the membranes were then blocked with 5% BSA and incubated with primary antibodies at 4 °C overnight. Next, the membranes were washed three times and then incubated with secondary antibodies for 1 h at room temperature. Quantitative densitometry was performed using ImageJ.

### 2.10 CRISPR/Cas9-mediated gene knockout

#### 2.10.1 Transfection

Single-guide RNAs were designed using the benchling.com website, and guides were cloned into pSpCas9(BB)-2A-Puro (PX459) V2.0 (gift from Jones laboratory). Human podocytes were plated in 6-well plates and transfected with 1 µg of guide-containing PX452 vectors. Cells transfected with a scrambled gRNA guide were used as the control (Supplementary Table S1A; Supplementary Figure S7). Transfected cells were selected with puromycin at 2 μg/mL starting 18 h after transfection and maintained for 48 h while changing the puromycin-containing media. Pooled KO cells were then amplified in regular media.

#### 2.10.2 TIDE analysis

Upon reaching confluence, genomic DNA was extracted from scrambled and KO cells using a QIAGEN Blood & Cell Culture DNA Kit, as per the manufacturer’s instructions. Regions flanking target sequences were amplified by PCR, with the primer sequences provided in Supplementary Table S1B. PCR products were then resolved on an agarose gel to verify amplification specificity. In cases where multiple bands were observed, amplicons of interest were isolated using a Monarch DNA Gel Extraction Kit (NEB, #T1020L). Amplified DNAs were subjected to Sanger sequencing. The resulting chromatograms were analyzed using the tracking of indels by decomposition (TIDE) tool (https://tide.nki.nl/) to determine the percentage of frameshift-inducing indels in KO pools compared to that in scrambled control cells. Knockout (KO) efficiency was found to be in the range between 62.1% and 91.7% (Supplementary Figure S6B).

### 2.11 RNA-seq analysis

Three podocyte scrambled controls and three *KIAA1522* KOs were generated using CRISPR guides listed in Supplementary Table S1C. KO efficiency was validated by immunoblotting (Supplementary Figure S8). RNA extraction was performed using an RNeasy Mini Kit from QIAGEN, Germantown, MD, USA. The reads were trimmed with fastp and aligned using the STAR aligner. Raw read counts were obtained with HTSeq. Batch effects were corrected using the sva R package. The DESeq2 R package was used to normalize counts and perform differential expression (DE) analysis between the conditions. Gene set enrichment analysis (GSEA) was performed using the clusterProfiler R package.

### 2.12 Immunofluorescence

Sixty thousand podocytes were cultured on collagen-coated coverslips in 12-well plates (collagen type I (Sigma)) and then differentiated at 37 °C for 5–7 days. Cells were fixed with 4% PFA in PBS and permeabilized using 0.5% Triton X-100 (Sigma-Aldrich, St. Louis, MO, USA) in PBS. Following blocking with 3% bovine serum albumin in PBS, cells were immunostained with the respective antibodies and stained with phalloidin. Images of the cells were taken using a Zeiss LSM 780, Oberkochen, Baden-Württemberg, Germany Laser Scanning Confocal Microscope. Cell size was quantified using ImageJ software. Vinculin count was quantified using the “analyze particle” command in ImageJ. Particle size was selected between 1 µm^2^–8 µm^2^. The filopodia localization ratio of KIAA1522 and IRSp53 was quantified using ImageJ software and calculated as (integrated density in the whole cell − integrated density in the inner cell body)/integrated density in the whole cell, following an approach analogous to the one we previously used to assess membrane localization of a protein (Matsuda et al., 2022).

#### 2.12.1 KIAA1522 immunostaining in podocytes

Podocytes were cultured in 12-well plates and transfected with 200 ng of GFP-Cdc42; constitutively active and dominant-negative vectors were overexpressed in podocytes. Cells were fixed and stained for KIAA1522. Images were taken using the Zeiss LSM 880 Laser Scanning Microscope using the structured illumination microscopy (SIM) technology.

#### 2.12.2 Live mCherry-paxillin imaging

Podocytes were cultured on a glass-bottom 32-mm dish coated with laminin and then transfected with mCherry-paxillin. Then, 24 h post-transfection, cells were imaged every 2 min for ∼60 min.

### 2.13 RhoA GLISA

Podocytes were either untreated or treated with EGF (100 ng/mL) for 5 min. Lysates were prepared, and GLISA was conducted according to the manufacturer’s instructions. Basal RhoA activity (unstimulated) of KO lines was calculated as the percentage of basal RhoA activity in the scrambled control.

### 2.14 Zebrafish maintenance and morpholino injections

Zebrafish AB and Tupfel long fin (TL) lines were used for *KIAA1522* and ARHGEF12 experiments, respectively. Zebrafish were maintained at 28.5 °C in E3 media (NaCl = 0.287 g/L, KCl = 0.0132 g/L, CaCl_2_·2H_2_O = 0.0479 g/L, MgCl_2_·6H_2_O = 0.0807 g/L, 0.00005% methylene blue, pH 7.2). Antisense morpholino oligonucleotides (MOs) were designed for translation blocking and purchased from Gene Tools (Gene Tools LLC, Philomath, OR, United States). Working MO dilutions were prepared in water (at 150 µM for *KIAA1522* and 250 µM for *arhgef12a + arhgef12b*) and heated at 65 °C for 5 min to disrupt potential secondary structures. Zebrafish embryos were microinjected into the yolk with the MO solution at the single-cell stage. Injected embryos were maintained in E3 media under standard conditions until 5 days post-fertilization (5 dpf). Live images were taken at 3 and/or 5 dpf while embryos were under anesthesia using Tricaine (168 mg/L).

#### 2.14.1 Morpholinos for *KIAA1522* experiments:

Scrambled control MO: random 25-base oligonucleotide mixture.

*kiaa1522* MO: 5'-AAAACACCACCATGTCTGTCTTGAG- 3′.

#### 2.14.2 Morpholinos for ARHGEF12 experiments:

Standard control MO: 5'–CCTCTTACCTCAGTTACAATTTATA–3' (targeting human beta globulin).

*arhgef12a* MO: 5′- TGACTGTAGACCGTGTGTCGCTCAT - 3′.

*arhgef12b* MO: 5′- CACCAGTCTGAACACCAGCTCGCAT - 3′.

### 2.15 Transmission electron microscopy

Zebrafish were fixed in 2% glutaraldehyde. Transmission electron micrographs of glomerular tufts were captured using a 120 kV Hitachi H-7650 Transmission Electron Microscope (Hitachi, Tokyo, Japan).

### 2.16 Antibodies and reagents

The antibodies and reagents are listed in Supplementary Table S2.

## 3 Results

### 3.1 Rho GTPase interactome is determined by their GTP-loading status and cell type

**FIGURE 1:**
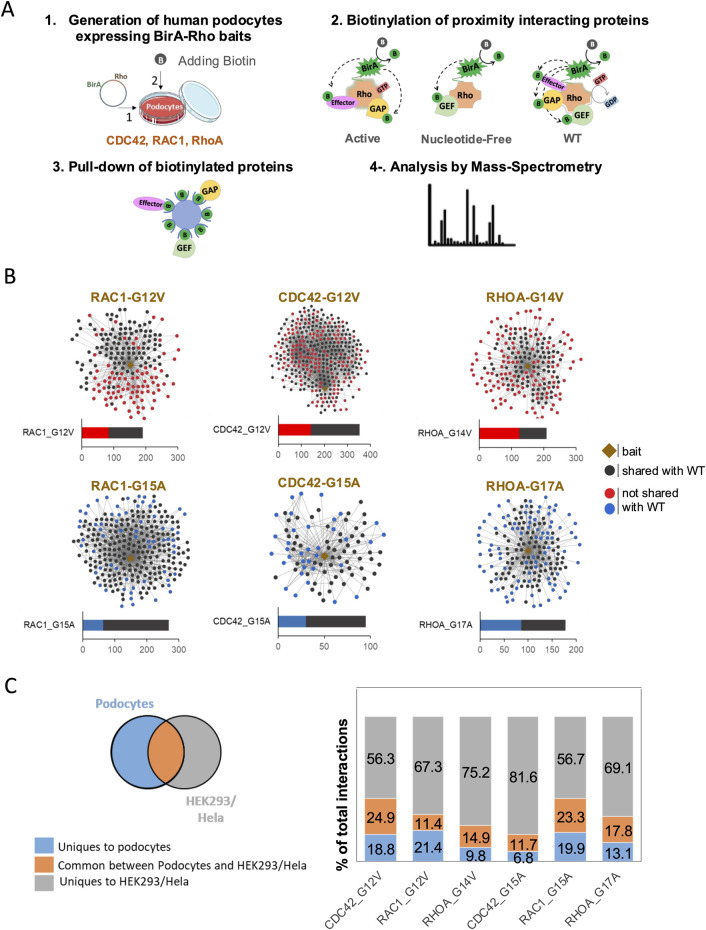
BioID experimental design and Rho GTPase interaction networks in podocytes. **(A)** Experimental design of BioID workflow in podocytes. (1) Podocytes expressing inducible BirA-Rho GTPase vectors (Rac1, Cdc42, and RhoA) were generated. Biotin was added to enable BirA-mediated biotinylation of proximity prey proteins. (2) Three types of baits were used: active baits (Rac1-G12V, Cdc42-G12V, and RhoA-G14V) that are GTP-bound, nucleotide-free or NF (Rac1-G15A, Cdc42-G15A, and RhoA-G17A), and WT (Rac1, Cdc42, and RhoA). (3) Biotinylated proteins were pulled down using streptavidin beads. (4) Mass spectrometry was performed to identify the biotinylated proteins. **(B)** Interaction networks showing the identified preys of active and NF baits. Black dots indicate preys shared with WT baits, while red and blue dots, respectively, indicate preys that are unique to active or NF baits compared to the corresponding WT baits. **(C)** Venn diagram and histogram summarizing the comparison of Rho GTPases preys identified in podocytes with those detected in HEK293 and HeLa cells (Bagci et al., 2020).

To map the interactome of Rho GTPase in human podocytes, we performed BioID analysis in *in vitro*-cultured immortalized podocytes. We used baits representing different nucleotide loading statuses of the three prototypical Rho GTPases: the active, GTP-bound forms (RhoAG14V, Rac1G12V, and Cdc42G12V), the NF forms that bind GEFs with high affinity (RhoAG17A, Rac1G15A, and Cdc42G15A), and WT, which cycles between GTP- and GDP-bound forms. The nine baits revealed 1,927 significant protein–protein interactions in podocytes (Supplementary Table S3). We first compared the hits captured by the active baits with those from WT baits and found that 40%–59% of the interactions were unique for active baits (Figure 1A). Similarly, comparing the hits captured by the NF baits to those from WT baits revealed that 24%–48% of NF baits’ interactions were unique (Figure 1B). Thus, our approach of using baits with distinct nucleotide loading statuses allowed the sensitive detection of specific interactors, enabling the discovery of distinct groups of interactors, including potential activators, inactivators, and effectors.

To assess the cell-type specificity of Rho GTPase interactions, we next compared our results to previously published datasets from HEK293 and HeLa cells using a similar BioID approach (Bagci et al., 2020). Among the six baits (active and NF), 11%–25% of all the hits were common between podocytes and HEK293/HeLa cells, 7%–21% of the hits were unique to podocytes, and 56%–82% of the hits were unique to HEK293/HeLa cells (Figure 1C; Supplementary Table S4). Notably, approximately 50% of the Rho GTPase interactors identified in podocytes were absent in HEK293 and HeLa cells. This suggests the existence of podocyte-specific Rho GTPase regulatory mechanisms and signaling networks.

### 3.2 Active bait interactors are enriched for cytoskeletal and morphological functions

**FIGURE 2:**
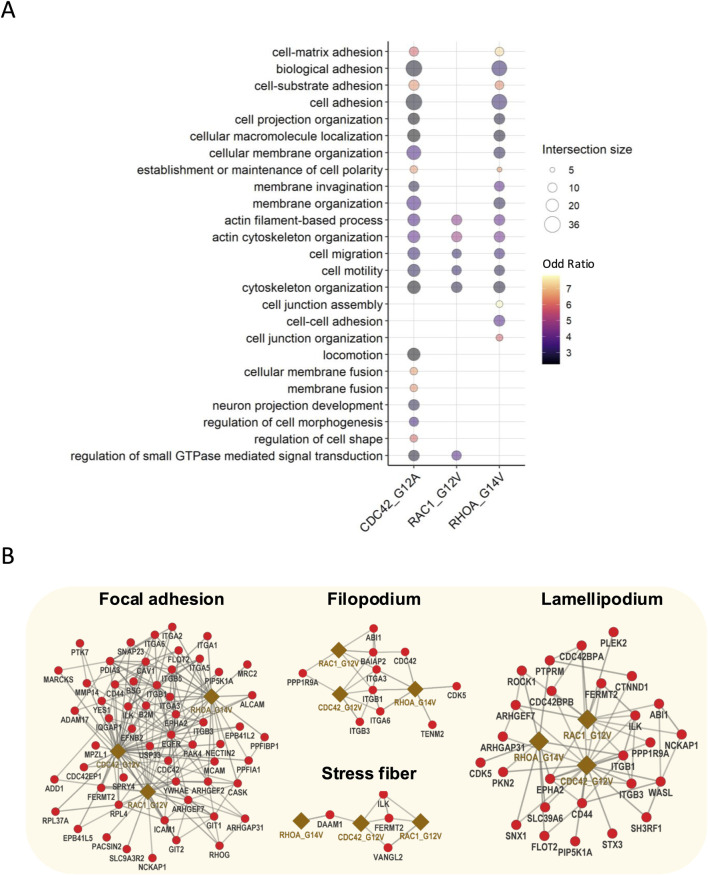
Gene Ontology enrichment and interaction networks of active bait interactors. **(A)** GO enrichment analysis of active bait interactors highlighting the top 25 enriched biological processes related to cell morphology and cytoskeleton regulation. **(B)** Interaction network active baits and effector proteins involved in the formation and the regulation of lamellipodia, focal adhesion, stress fibers, and filopodia.

To gain insights into the functional roles of active bait interactors, we next performed Gene Ontology (GO) enrichment analysis as these interact or are expected to interact with downstream effectors. Our analysis revealed a significant enrichment of GO biological processes (GO-BP) related to cell morphology and cytoskeleton regulation, including cell–matrix interaction, actin filament-based process, and cell–cell adhesion (Figure 2A; Supplementary Figure S1A). Further analysis of the GO cellular components (GO-CC) indicated that these interactors are enriched in key pathways involved in cellular structures such as stress fiber, lamellipodium, filopodium, and focal adhesion (Figure 2B; Supplementary Figure S1B). Moreover, the identified hits included known interactors of Rho GTPases. For instance, active RhoA interacts with ROCK1, ROCK2, DIAPH2, and DIAPH3, which are essential for stress fiber formation (Shi et al., 2013; Watanabe et al., 1999). Similarly, active Rac1 interacted with ABI1, NCKAP1, and CYFIP2, which are key components in WAVE complexes and crucial for lamellipodia formation (Rottner et al., 2021). Furthermore, active Cdc42 identified WIPF1, WIPF2, WASL, and BAIAP2, all of which contribute to filopodia formation (Derivery and Gautreau, 2010) (Supplementary Table S3). In addition, by using WD scores to measure the hit specificity, we identified the top 30 interactors for each active bait, revealing potentially critical components and effectors of their respective signaling pathways (Supplementary Figure S2).

Together, these results uncover the interactomes of Rho GTPases in human podocytes and suggest that Rho GTPase signaling in podocytes is closely linked to cytoskeletal dynamics. Our findings are consistent with established Rho GTPase functions, further validating the robustness of our approach.

### 3.3 KIAA1522 is a potential effector of Cdc42 in podocytes involved in filopodia formation

**FIGURE 3:**
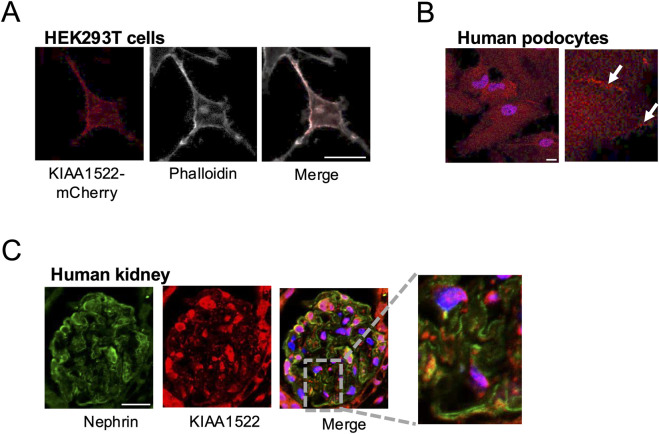
Expression pattern of KIAA1522 in podocytes. **(A)** Immunofluorescence staining showing partial co-localization between KIAA1522 (red) and podocyte marker nephrin (green) in human glomerulus. **(B)** Confocal images showing co-localization between KIAA1522 and F-actin (phalloidin staining) in HEK293 cells overexpressing KIAA1522-mCherry. **(C)** KIAA1522 immunofluorescence staining in human podocytes shows increased localization (arrows) at the cell membrane and cell–cell junctions (bars 20 μm).

Our BioID results identified KIAA1522, a protein with a minimally characterized function, as an interactor of active Rac1 and Cdc42. Since the role of KIAA1522 in cytoskeletal regulation had not been explored, we sought to investigate its function in podocytes. First, we investigated the expression pattern of KIAA1522. The overexpression of mCherry-tagged KIAA1522 in HEK293 cells revealed membrane localization and co-localization with F-actin (phalloidin) (Figure 3A). Immunofluorescence staining of KIAA1522 in immortalized human podocytes also showed a clear localization at the plasma membrane, particularly at cell–cell junctions (Figure 3B). In addition, immunofluorescence staining of human kidney sections showed that KIAA1522 is expressed in the glomerulus, with partial co-localization with the podocyte-specific transmembrane protein nephrin (Figure 3C).

**FIGURE 4:**
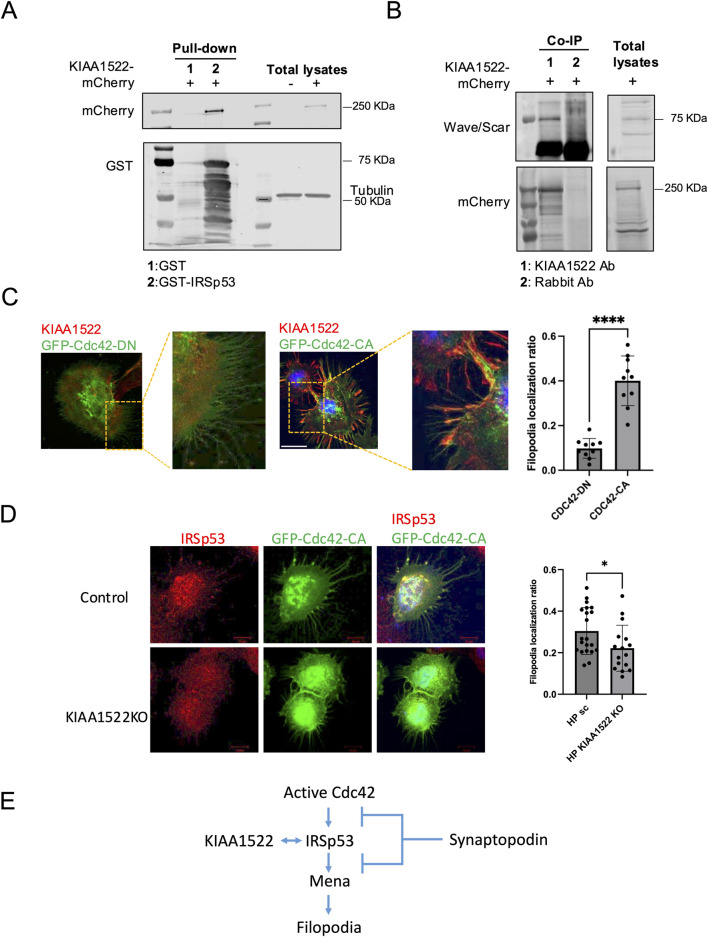
KIAA1522 functions as a Cdc42 effector involved in filopodia. **(A)** Immunoblotting for mCherry following a GST pull-down assay using GST-IRSp53 beads incubated with lysates from HEK293 cells overexpressing KIAA1522-mCherry. **(B)** Immunoprecipitation of KIAA1522 from HEK293 cell lysates overexpressing KIAA1522-mCherry, followed by immunoblotting for scar/wave complex proteins. **(C)** Structured illumination microscopy (SIM) images of podocytes overexpressing GFP-Cdc42-DN and GFP-Cdc42-CA (left panel). Filopodia localization of KIAA1522 was quantified as described in section 2.12 (right panel). (Bars 20 μm; t-test, **** *p*-value <0.001) **(D)** Confocal microscopy images of control and *KIAA1522* KO podocytes transfected with GFP-Cdc42-CA. Filopodia localization of IRSp53 was quantified as described in Section 2.12 (bars 10 μm; t-test, * *p*-value <0.05). **(E)**. Proposed pathway of filopodium formation, based on Yanagida-Asanuma et al., 2007.

To identify potential interacting partners of KIAA1522 and gain mechanistic insight into its role, we searched the BioGRID database for potential KIAA1522 interactors predicted by Affinity Capture-MS (https://thebiogrid.org/). BioGRID identified IRSp53, a key adaptor protein involved in filopodia formation (Krugmann et al., 2001), and WAVE/SCAR, a Rac1 effector implicated in lamellipodia formation (Abou Kheir et al., 2005), as putative interactors of KIAA1522. We next performed a pull-down assay, which confirmed the interaction between KIAA1522 and IRSp53 (Figure 4A). Immunoprecipitation further demonstrated that KIAA1522 interacts with WAVE/SCAR (Figure 4B). This suggests that KIAA1522 may play an active role in the dynamics of the actin cytoskeleton.

Notably, our BioID data showed that, similar to KIAA1522, IRSp53 interacted with Cdc42 and Rac1 (Supplementary Table S3). IRSp53 promotes filopodia formation by acting as the Cdc42 effector, where the latter causes conformational changes to IRSp53, enabling Mena recruitment and subsequent actin filament assembly (Krugmann et al., 2001). In podocytes, the formation of Cdc42: IRSp53: Mena complexes was found to be suppressed by synaptopodin, which is a key protein known for its role in maintaining podocyte integrity and protecting against proteinuria (Yanagida-Asanuma et al., 2007). To investigate whether KIAA1522 acts as a Cdc42 effector in podocytes, we examined whether the Cdc42 activation status induced its specific recruitment. For this, we overexpressed active and dominant-negative forms of Cdc42 in podocytes and assessed KIAA1522 localization by immunofluorescence. When the active Cdc42 was expressed, KIAA1522 was specifically recruited to Cdc42-induced filopodia. In contrast, when the dominant-negative form of Cdc42 was expressed, KIAA1522 was strictly localized in the cytoplasm (Figure 4C). These results suggest that KIAA1522 may function in the filopodium complex at the plasma membrane downstream of Cdc42, possibly via IRSp53. To test this further, we used pooled CRISPR KO podocyte lines (see section 2.11). When *KIAA1522* KO podocytes were transfected with active Cdc42, IRSp53 localization to filopodia was reduced significantly compared to that of the control podocytes (Figure 4D). These findings support that KIAA1522 interacts with IRSp53 and likely facilitates its relocalization to the plasma membrane, thereby contributing to filopodium formation (Figure 4E).

**FIGURE 5:**
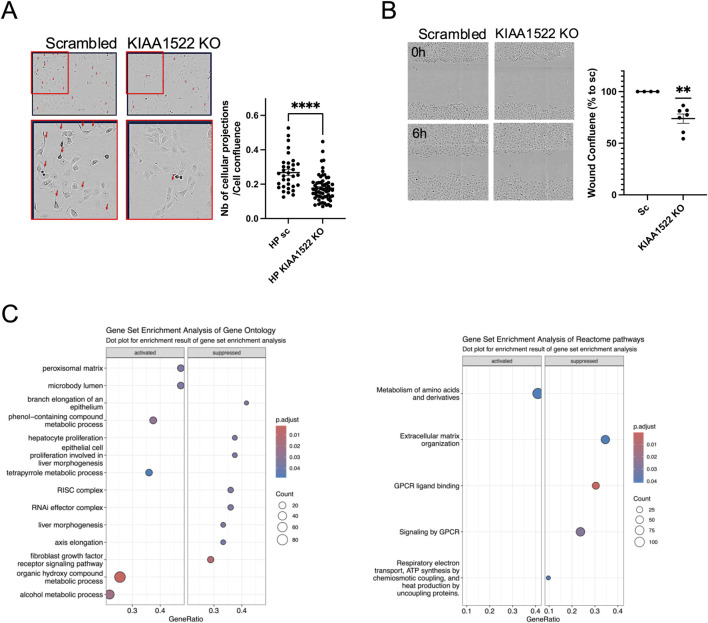
KIAA1522 regulates cytoskeletal dynamics in podocytes. **(A)** Representative images (left panel) and quantification (right panel) of cellular projections (Arrows) induced by EGF in scrambled control and *KIAA1522* KO podocytes, normalized to the percentage of cell confluence (t-test, **** *p*-value <0.001). **(B)** Representative wound healing assay images (left panel) and quantifications of wound confluence at 6 h showing reduced migration in *KIAA1522* KO cells than in controls (one simple t-test, ** *p*-value <0.01). **(C)** Bubble plots summarizing the altered pathways found by GSEA of GO pathways (left panel) and Reactome pathways (right panel) in *KIAA1522* KO podocytes compared to control.

We next examined the response of *KIAA1522* KO podocytes to EGF stimulation, which activates Rho GTPases and induces cell motility. *KIAA1522* KO podocytes exhibited a reduced cell projection formation following EGF treatment (Figure 5A). In addition, *KIAA1522* KO significantly decreased podocyte migration, with an average migration rate of 74% compared to that in the control (*p*-value = 0.0011; Figure 5B). Together, these findings confirm that KIAA1522 plays a role in cytoskeletal regulation and podocyte motility. Next, to investigate the signaling pathways associated with KIAA1522, we performed RNA sequencing of *KIAA1522* KO HPs and controls. While differential expression (DE) analysis showed significant but modest changes at the gene level (Supplementary Tables S5, S6), we utilized GSEA to identify broader pathway-level alterations. GSEA of GO pathways revealed a downregulation of genes associated with the RNAi effector complex pathway and the RISC complex compared to that in the control (Figure 5C, left panel). In addition, GSEA of the Reactome pathways showed a downregulation of genes involved in extracellular matrix organization (Figure 5C, right panel). These findings suggest that KIAA1522 may influence post-transcriptional regulation and play a role in regulating cell motility, likely by modulating actin rearrangement, promoting filopodia and cell projection formation, and indirectly affecting the extracellular matrix composition.

**FIGURE 6:**
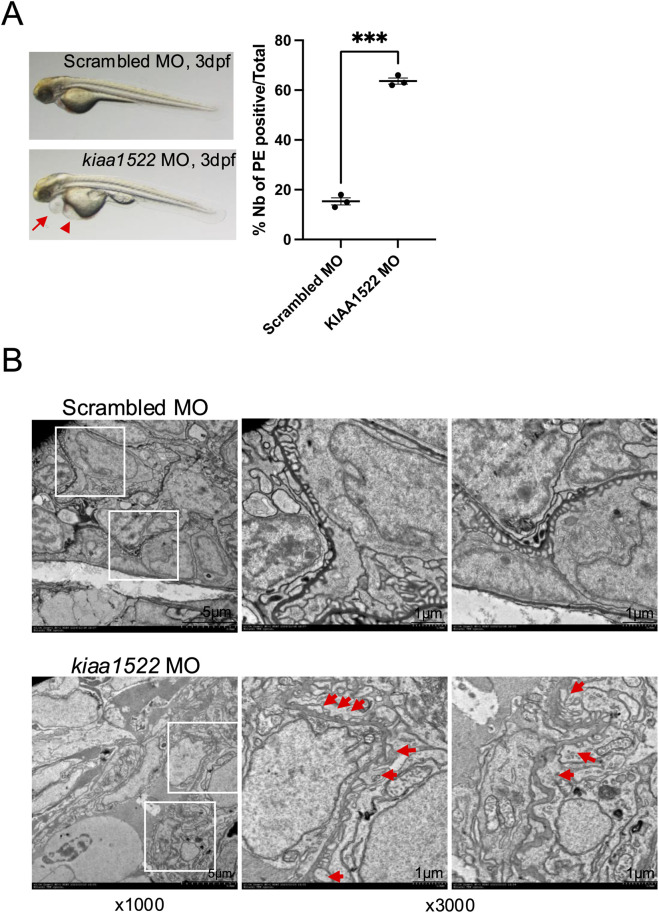
*KIAA1522* knock-down disrupts the glomerular function in zebrafish. **(A)** Representative images of control and *KIAA1522* morphant zebrafish at 3 dpf showing pericardial edema (PE) (arrow) and yolk sac edema (arrowhead) in *KIAA1522* morphants (left panel). Percentage of occurrence of PE in *KIAA1522* zebrafish morphants compared to that in control morphants (right panel) (paired t-test, *** *p*-value <0.001). **(B)** TEM images of the glomerular filtration barrier of the control and *KIAA1522* morphants showing foot process effacement (arrows) in *KIAA1522* morphants (bars 1 μm).

To investigate the functional role of KIAA1522 *in vivo*, we transiently knocked down its homolog in zebrafish larvae, a well-established model for studying podocyte biology and screening for proteinuric phenotypes (Schindler and Endlich, 2024). Between 62% and 65% of *KIAA1522* morphants (n = 69) exhibited pericardial effusion/edema starting at 3 dpf compared with 13%–18% of control morphants (n = 46). Yolk sac edema was also observed in *KIAA1522* morphants (Figure 6A). This pericardial edema phenotype may indicate proteinuria as similar phenotypes have been observed following knockdown of proteins critical for glomerular function, including nephrin, podocin, and Apol1 (Fukuyo et al., 2014; Kotb et al., 2016; Lee et al., 2022). Notably, pericardial edema has also been reported in the metronidazole-induced podocyte injury model (Siegerist et al., 2017). Next, we analyzed the ultrastructure of the glomerular filtration barrier of these morphants by transmission electron microscopy (TEM). We identified significant foot process effacement of podocytes in *KIAA1522* morphants compared to that in the control, indicating podocyte injury and subsequent disruption of the glomerular function (Figure 6B). Foot process effacement is a biomarker of proteinuric diseases, and thus, our findings reveal that *KIAA1522* is crucial for maintaining podocyte integrity and normal glomerular function.

### 3.4 Interactions of Rho GTPases with their regulatory proteins GAPs and GEFs

Rho GTPase activities are regulated by three groups of proteins: 1) activator GEFs (82 members), 2) inactivator GAPs (69 members), and 3) guanine nucleotide dissociation inhibitors (GDIs), which bind to the GDP-bound inactive forms, maintaining a pool of inactive Rho GTPases (three members). The interplay between these numerous GEFs/GAPs and the 21 Rho GTPase members creates the complexity of the Rho GTPase signaling network. To dissect this network in podocytes, we analyzed our BioID results, focusing on GEFs and GAPs. The NF baits are known to bind to GEFs with high affinity and are commonly used to identify GEFs for a specific Rho GTPase, whereas the active baits are more likely to interact with GAPs. Additionally, WT control baits are expected to interact with GEFs and GAPs at lower affinity than their mutated counterparts.

**FIGURE 7:**
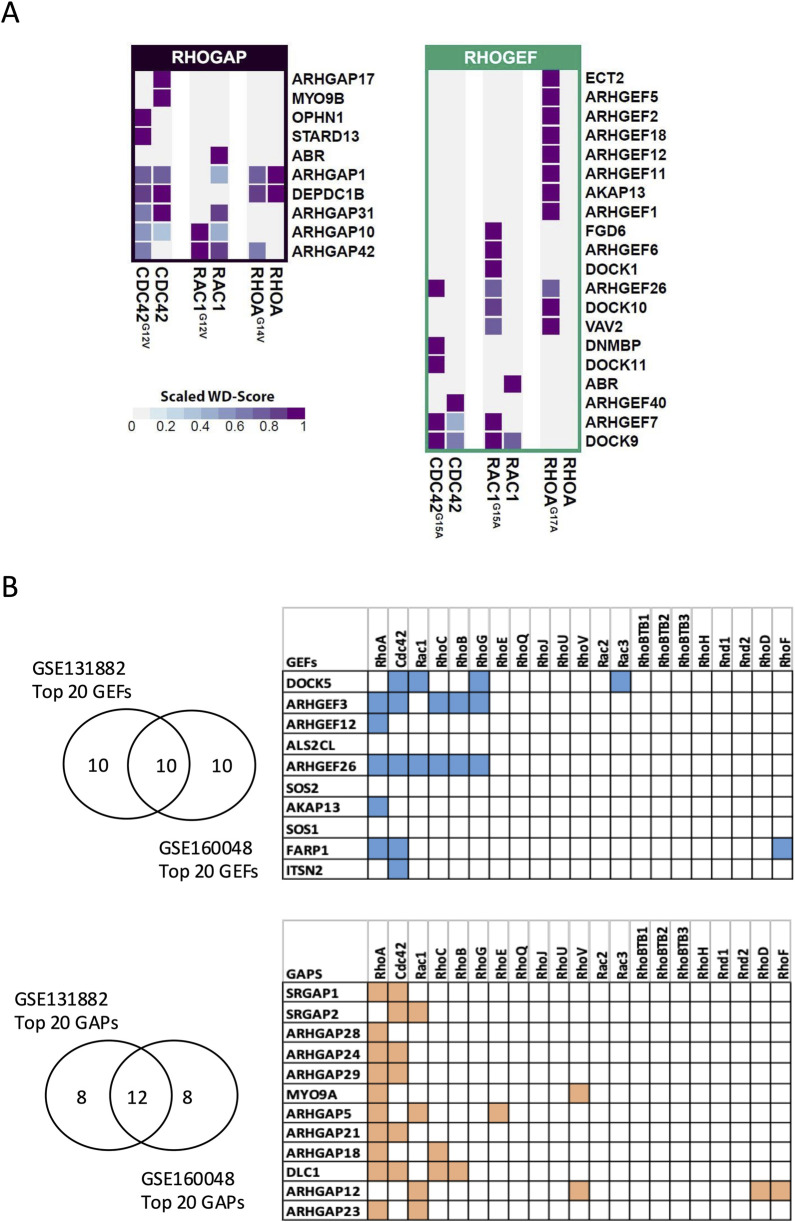
Identification of GEFs and GAPs in podocytes through BioID and transcriptomic analysis **(A)** and by public transcriptomic analysis **(B)**. **(A)** Heatmaps showing GAPs (left) and GEFs (right) detected by BioID, along with their relative specificity (scaled WD score) of interactions with WT, active, and NF baits (right). **(B)** Top highly expressed GEFs and GAPs in human podocytes, along with their corresponding Rho GTPases based on the STRING database. The top 20 GEFs and GAPs by expression level were first extracted from two independent transcriptomic datasets of human podocytes (GSE131882 and GSE160048). Only genes ranked within the top 20 in both datasets were retained for analysis.

Our results showed that NF-RhoA (RhoAG17A) interacted with 11 GEFs, while NF-Rac1 (Rac1G15A) and NF-Cdc42 (Cdc42G15A) interacted with 8 and 5 GEFs, respectively. Although some GEFs exhibited specificity for a single Rho GTPase, others were promiscuous. Specifically, eight GEFs were unique to RhoA (ECT2, ARHGEF5, ARHGEF2, ARHGEF18, ARHGEF12, ARHGEF11, AKAP13, and ARHGEF1), two were specific to Cdc42 (DNMBP and DOCK11), and three were specific to Rac1 (FGD6, ARHGEF6, and DOCK1). In contrast, VAV2 interacted with both RhoA and Rac1, ARHGEF7 and DOCK9 were shared between Rac1 and Cdc42, and ARHGEF26 interacted with all three Rho GTPases (Figure 7A, right panel). Notably, the majority of the identified GEFs interacted exclusively with the NF forms, which is consistent with previous reports showing that the NF forms have a high affinity for GEFs. On the other hand, ABR and ARHGEF40 interacted only with WT-Rac1 and WT-Cdc42, respectively, although the reason and significance of this unexpected interaction remain unclear. The GAPs detected with the active baits were fewer in number than the GEFs detected with the NF baits, and they were found to interact with the WT forms. In addition, GAPs were less specific to a single Rho GTPase, with five GAPs interacting specifically with one Rho GTPase and five others interacting with multiple Rho GTPases (Figure 7A, left panel). Overall, the BioID results identified the panel of GEFs and GAPs that interact with RhoA, Rac1, and Cdc42 in human podocytes.

### 3.5 Transcriptomic screening of Rho GTPases and their regulators highlights the dominant role of the RhoA pathway in primary podocytes

BioID experiments yielded numerous GEFs and GAPs as potential interactors of Rho GTPases in podocytes. To refine the focus of our study, we analyzed publicly available transcriptomic databases to assess the expression profile of GEFs and GAPs in human podocytes. First, we examined the expression of 21 Rho GTPases, 69 Rho GAPs, and 82 Rho GEFs in two independent single-cell and single-nucleus RNA sequencing (scRNA-seq and snRNA-seq) datasets from healthy kidney tissues. In both datasets, podocytes expressed the three prototypical Rho GTPases (RHOA, RAC1, and Cdc42) along with two additional members, RHOQ and RHOBTB2 (Supplementary Figure S3). Among the top 20 most abundantly expressed GEFs in podocytes from each dataset, 10 were common in both datasets. Similarly, 12 GAPs were commonly ranked among the top 20 most abundant GAPs in both datasets (Figure 7B, left panel; Supplementary Figures S4, S5). To identify the dominant Rho GTPase signaling pathways in podocytes, we analyzed the interaction of the identified 10 GEFs and 12 GAPs with a comprehensive panel of Rho GTPases using the STRING database. Our analysis revealed that six or more of these GEFs and GAPs interacted with RhoA, Cdc42, and Rac1, which represented the most frequent interactions (Figure 7B, right panel).

Specifically, RhoA interacted with five GEFs and ten GAPs, Cdc42 interacted with five GEFs and six GAPs, and Rac1 interacted with two GEFs and four GAPs. Notably, two RhoA GEFs (ARHGEF3 and ARHGEF26) also interacted with RhoB and RhoC (which are structurally similar to RhoA) (Wheeler and Ridley, 2004) and with RhoG (which resembles Rac1 and Cdc42). Overall, the high abundance of RhoA modulators suggests that RhoA is a major Rho GTPase in healthy human podocytes.

Next, to determine whether the expression levels of identified GEFs and GAPs are altered in human podocytes under kidney disease conditions, we analyzed the snRNA-seq dataset from Wilson et al. (2019), which compares proteinuric and diabetic kidneys to healthy controls. Notably, the RhoA-targeting GAP, DLC1, was significantly upregulated in podocytes from diabetic kidneys (log2 FC: 0.39, *p*-value = 1.01E-09). Moreover, DLC1 expression was significantly higher in podocytes from proteinuric DKD patients than in those from non-proteinuric DKD patients (log2 FC = 0.56, *p*-value = 2.1E-07). Although the significance of these changes remains to be determined, they support the notion that regulation of RhoA activity in podocytes is crucial in both health and disease.

### 3.6 Screening of RhoA GEF functions in podocytes and the role of ARHGEF12 in podocyte motility and integrity

Based on the BioID results and database analysis, we proceeded to screen the function of 11 RhoA-targeting GEFs identified by BioID in human podocytes *in vitro.* Using CRISPR/Cas9, we generated pooled KO lines for each RhoA-GEF gene in immortalized human podocytes (Supplementary Figure S6A, B).

We first studied the impact of each KO on basal RhoA activity using GLISA, which revealed that the knockout of 9 out of 11 RhoA GEFs reduced basal RhoA activity. Among these, *ARHGEF12* KO had the most pronounced effect on RhoA activity, followed by *ARHGEF1* KO and *ARHGEF11* KO (Supplementary Figure S6C). In addition, the depletion of *VAV2*, *DOCK10*, *ARHGEF12*, *ARHGEF11*, and *ARHGEF1* resulted in reduced cell size (Supplementary Figure S6D).

Interestingly, analysis of a healthy human kidney dataset from the Broad Institute’s Single-Cell Portal revealed that ARHGEF12 and ARHGEF26 are more specifically enriched in podocytes than in other kidney cell types (Supplementary Figure S6E). In addition, ARHGEF12 had the highest average spectral counts (AvgSpec) among the RhoA GEFs interacting with NF RhoA in our BioID results (Supplementary Figure S6B). ARHGEF12 was also among the most highly expressed GEFs in podocytes from the two independent primary single-cell transcriptomics datasets examined above (Figure 7B).

**FIGURE 8:**
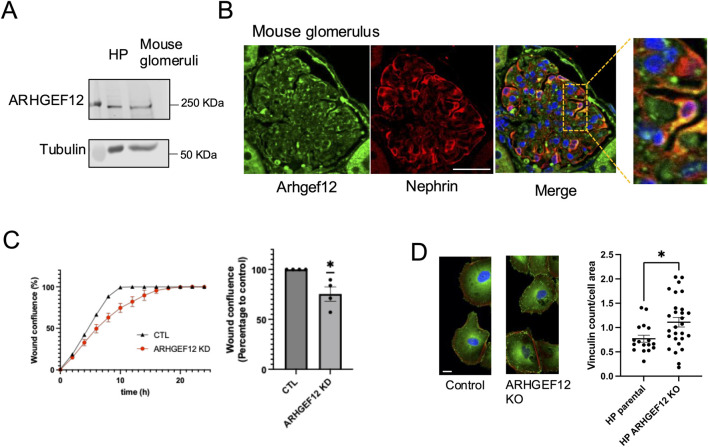
Role of ARHGEF12 in the regulation of podocyte focal adhesion and cell motility. **(A)** Western blot showing ARHGEF12 protein expression in mouse glomeruli. **(B)** Immunostaining showing partial co-localization of ARHGEF12 with nephrin in mouse glomeruli. **(C)** Representative wound confluence curves (left) showing wound closure in ARHGEF12 KD podocytes compared to that in the control, alongside a plot showing the percentage of wound confluence to the control at 8 h of separate experiments (one simple t-test, * *p*-value <0.05). **(D)** Immunostaining of vinculin in *ARHGEF12* KO podocytes compared to that in the control (left), and quantification of vinculin count per cell area (right) normalized to the cell area (t-test, * *p*-value <0.05, bars 20 μm).

Given its prominent expression profile and activity, we chose to further investigate the role of ARHGEF12 in podocytes. First, we validated ARHGEF12 protein expression by immunoblotting in human podocytes *in vitro* and mouse glomerular lysates (Figure 8A). Immunofluorescence staining confirmed ARHGEF12 expression in the mouse glomerulus, with partial co-localization observed between ARHGEF12 and nephrin in podocytes (Figure 8B). Functionally, *ARHGEF12* KO cells exhibited a reduced migration rate, as determined by the wound healing assay (Figure 8C).

In addition, *ARHGEF12* KO cells displayed an increase in vinculin complex number per cell area, indicating that ARHGEF12 may play a role in the turnover and/or maturation of focal adhesion complexes (Figure 8D). This increase in focal adhesion at both the leading and trailing edges likely impairs cell motility by preventing the cells from forming new complexes or detaching properly. Live imaging of podocytes transfected with mCherry-paxillin further demonstrated impaired cell motility and revealed the abundance of small and persistent focal adhesions in *ARHGEF12* KO cells compared to those in the control (Supplementary Videos S1, S2).

**FIGURE 9:**
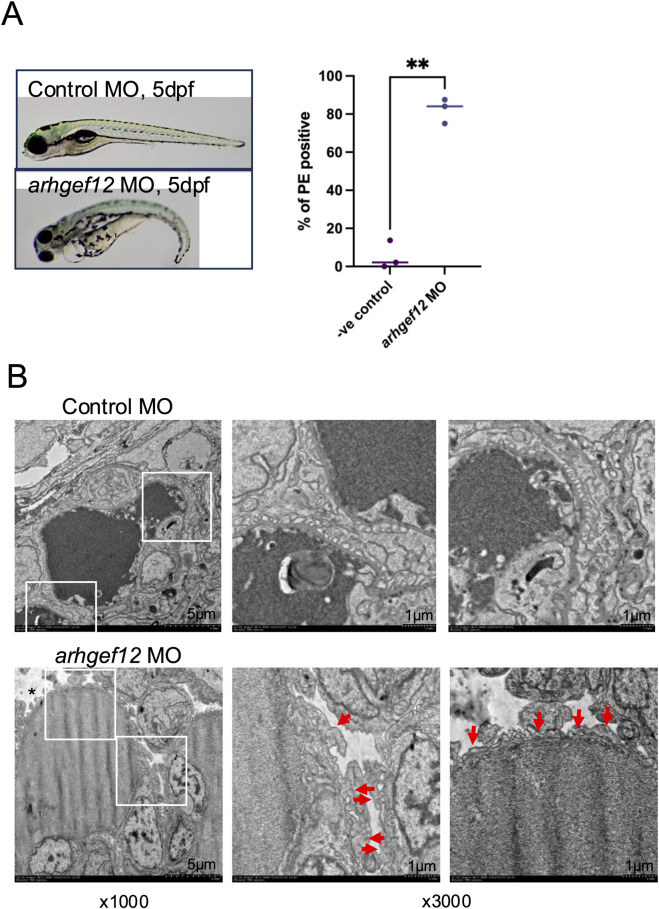
ARHGEF12 knock-down disrupts the glomerular function in zebrafish. **(A)** Representative images of control and ARHGEF12 morphants at 5 dpf showing pericardial edema (arrow) in ARHGEF12 morphants (left panel). Percentage of occurrence of pericardial edema (PE) in ARHGEF12 morphants compared to that in control morphants (right panel) (paired t-test, ** *p*-value <0,01). **(B)** TEM images of the glomerular filtration barrier of the control and ARHGEF12 morphants showing foot process effacement (arrows) and edema (asterisk) in ARHGEF12 morphants (bars 1 μm).

Finally, to investigate the functional role of ARHGEF12 *in vivo*, we transiently knocked-down *ARHGEF12* homologs (*arhgef12a* and *arhgef12b*) in zebrafish larvae. This induced pericardial edema in zebrafish at 5 dpf in 75%–87% ARHGEF12 morphants (n = 190) compared with 2%–14% of control morphants (n = 182) (Figure 9A). Analysis of the ultrastructure of the glomerular filtration barrier by TEM revealed foot process effacement of podocytes in ARHGEF12 morphants compared to that in the control (Figure 9B). Together, these results indicate that ARHGEF12 is the main RhoA-GEF in podocytes and is crucial for their integrity and motility.

## 4 Discussion

In this study, for the first time, we dissected the upstream regulators and the downstream effectors of Rho GTPases in podocytes. We implemented BioID for its ability to capture transient protein–protein interactions in a near-physiological context and identify interactors across various cellular compartments, offering advantages over traditional protein-interaction techniques (Kim and Roux, 2016; Roux et al., 2018). Our BioID results provide a comprehensive interaction network of Rho GTPase regulators in podocytes. In addition to the panel of potential effector proteins, our analysis revealed aspects of specificity and cross-talk of Rho GTPase regulatory proteins. These observations align with the complexity of Rho GTPase regulation, where a limited number of Rho GTPases are controlled by a large network of regulatory proteins. Our study benefits from using immortalized human podocytes, a well-established *in vitro* model of podocyte function and signaling. These cells, however, lack the characteristic three-dimensional morphology of podocytes, such as foot processes and the slit diaphragm. Thus, while not fully representative of *in vivo* conditions, our novel interactome results offer a foundation for further exploration in more physiologically relevant models. Specifically, using podocytes cultured in 3D models or induced pluripotent stem cell (iPSC)-derived kidney organoids could provide broader insights into Rho GTPase interactions and signaling in foot process structures and help address the knowledge gap in the temporal and spatial activities of Rho GTPases in podocytes.

We identified KIAA1522 as a novel Cdc42 effector that participates in filopodia formation. KIAA1522 had been minimally characterized, and its precise function and interactome were largely unknown. Previous studies identified KIAA1522 as a prognostic biomarker across multiple cancers, where it was linked to tumor progression, therapy resistance, and poor survival (Li et al., 2023; Liu et al., 2016; Wang et al., 2020; Xu et al., 2020). KIAA1522 has been reported to promote cancer proliferation, migration, invasion, and metastasis through key pathways such as Wnt/β-catenin, ERK, Notch, and various lncRNA/circRNA–microRNA axes (Fan et al., 2022; Guo et al., 2020; Hu et al., 2021; Jiang et al., 2020; Lin et al., 2021; Xie et al., 2017; Yi et al., 2022). Conversely, tumor suppressors such as miR-378h, miR-125b-5p, and KLF9 were shown to inhibit KIAA1522 expression, highlighting its potential as a central therapeutic target across diverse malignancies (Guo et al., 2024; Lee et al., 2023; Li et al., 2018). Our work revealed, for the first time, an implication of KIAA1522 in the cytoskeleton dynamics of podocytes and specifically downstream of Cdc42. Although further studies in mice are needed to establish the functional role of KIAA1522 in podocytes, our results demonstrate that BioID is a powerful tool for identifying novel effector proteins regulating podocyte function.

We also identified RhoA as a major Rho GTPase in podocytes. RhoA, as a key member of the Rho GTPase family, plays a strongly controlled role in podocyte morphology and function. While the reduced activity of RhoA in podocytes can lead to the destabilization of the cytoskeleton, foot process effacement, and podocyte apoptosis (Huang et al., 2016; Wang et al., 2012), studies from our laboratory and others have shown that its excessive activation can also be detrimental to podocyte health (Peng et al., 2008; Zhu et al., 2011). These studies provide evidence that the alteration of RhoA activity contributes to the pathogenesis of proteinuric kidney diseases, highlighting the importance of studying RhoA regulators in podocytes.

DLC1 (deleted in liver cancer 1) is a Rho GTPase-activating protein that negatively regulates RhoA by promoting its GTP hydrolysis, thereby modulating actin cytoskeleton remodeling in various cell types (Joshi et al., 2020; Shih et al., 2015). Overexpression of DLC1 has been shown to reduce RhoA activity, whereas its knockdown results in increased RhoA activation (Matsuda et al., 2021). In diabetic podocytes, elevated DLC1 expression likely contributes to RhoA inactivation, disrupting RhoA-dependent processes such as cytoskeletal organization and foot process integrity. Since podocytes rely on strongly regulated RhoA signaling to preserve their complex architecture—including foot processes and slit diaphragms—DLC1 upregulation may destabilize the glomerular filtration barrier and promote proteinuria in diabetic kidney disease (Rachubik et al., 2022). Conversely, several studies have shown that excessive RhoA activation is also detrimental in diabetic nephropathy and that its inhibition may offer therapeutic benefits (Kolavennu et al., 2008; Komers, 2013). In this context, increased DLC1 expression may reflect an adaptive mechanism aimed at counteracting pathological RhoA hyperactivation.

Our results uncovered ARHGEF12 as a key RhoA regulator involved in podocyte motility. The pronounced impact of *ARHGEF12* KO on RhoA activity is likely attributed to its high abundance in podocytes. However, other factors, such as its subcellular localization and protein structure, could contribute to its prominent impact on RhoA activity and will be investigated in future studies. ARHGEF12, also known as leukemia-associated Rho GEF (LARG), has been implicated in the progression of various diseases, including cancer, pulmonary arterial hypertension, and pathogenic thrombus formation, primarily through its role in RhoA activation and its effects on the actin cytoskeleton (Ghanem et al., 2022). However, the role of ARHGEF12 in podocytes remained unclear. Previously, ARHGEF12 was found to interact with Wilms’ tumor interacting protein (WTIP). WTIP promotes actin stress fiber formation and focal adhesion maturation in a RhoA-dependent manner in cultured mouse podocytes (Kim et al., 2012). *In vivo*, WTIP localizes at the foot processes and plays a protective role against induced podocyte injury (Madhavan et al., 2022). However, the functional relationship between WTIP and ARHGEF12 in regulating RhoA activity, actin cytoskeletal dynamics, and podocyte phenotype has not been investigated. To our knowledge, our work shows, for the first time, using multiple lines of evidence, that ARHGEF12 is a key RhoA regulator in podocytes. In addition, ARHGEF12 is highly specific to podocytes within the kidney and is essential for maintaining normal podocyte motility and integrity.

In addition to its established role as a RhoA-GEF, ARHGEF12 was recently identified as a key mediator of RhoA–Rac1 crosstalk that regulates cell protrusion–retraction cycles (Nanda et al., 2023). Specifically, active Rac1 triggers the recruitment of ARHGEF12 to the plasma membrane at sites of cell protrusion via its PH domain, where ARHGEF12 activates RhoA. In turn, RhoA inhibits Rac1 activity, establishing a negative feedback loop that is essential for transitioning from protrusion to retraction. This dynamic regulation enables effective cell migration and precise positioning of cellular extensions, such as podocyte foot processes, and its alteration could impair podocyte integrity. In line with this, previous studies showed that both the increase and the decrease in podocyte motility in response to alteration of Rho GTPase regulators are linked to podocyte injury and subsequent proteinuria. For instance, the Rac1-GAP *ARHGAP24* knock-down in podocytes increased membrane ruffling, while a loss-of-function mutation in the *ARHGAP24* gene was associated with hereditary focal segmental glomerulosclerosis (FSGS), a kidney disease characterized by podocyte dysfunction (Akilesh et al., 2011). Similarly, mutations of the Rho GDP dissociation inhibitor α (GDIα) or *ARHGDIA*, known to cause congenital or infantile nephrotic syndrome, impaired coordinated movement and overall motility in podocytes (Auguste et al., 2016). Thus, ARHGEF12 may play an important role in maintaining podocyte structure and function by coordinating Rho GTPase activity.

By implementing zebrafish *in vivo* experiments, we showed that both *KIAA1522* and ARHGEF12 are important for the integrity of developing podocytes and the normal function of the glomerular filtration barrier. These findings validate our *in vitro* results and emphasize the relevance of our model in a physiological context, where podocyte ultrastructure is essential for proper the glomerular filtration barrier function. Future studies in mice could be devised to validate the observed phenotypes and characterize the role of these genes in disease progression.

Proteinuric kidney diseases are challenging conditions that often lead to kidney failure. While Rho GTPase alteration is proven to be pathogenic to podocyte health, Rho GTPases themselves are implicated in fundamental cellular functions throughout the body and are unlikely to be suitable as therapeutic targets. Thus, studying their interactors in podocytes may provide new insights into the mechanisms of proteinuria and help identify potential therapeutic targets, with the prospect of developing treatments that could improve the quality of life and prognosis of patients living with proteinuric kidney diseases.

## Data Availability

The original contributions presented in the study are included in the article/Supplementary Material, further inquiries can be directed to the corresponding author. The mass spectrometry proteomics data have been deposited to the ProteomeXchange Consortium via the PRIDE partner repository with the dataset identifier PXD069844, available at: https://www.ebi.ac.uk/pride/archive/projects/PXD069844.
