# Supplementary material for: Proximity-based proteomics (BioID) uncovers the Rho GTPase interactome in kidney podocytes
Source: Front Cell Dev Biol. 2025 Nov 11;13:1625950. doi: 10.3389/fcell.2025.1625950 (PMC12644026; doi:10.3389/fcell.2025.1625950)
Supplement: Supplementary file 3 [file Presentation1.pptx]

## Slide 1
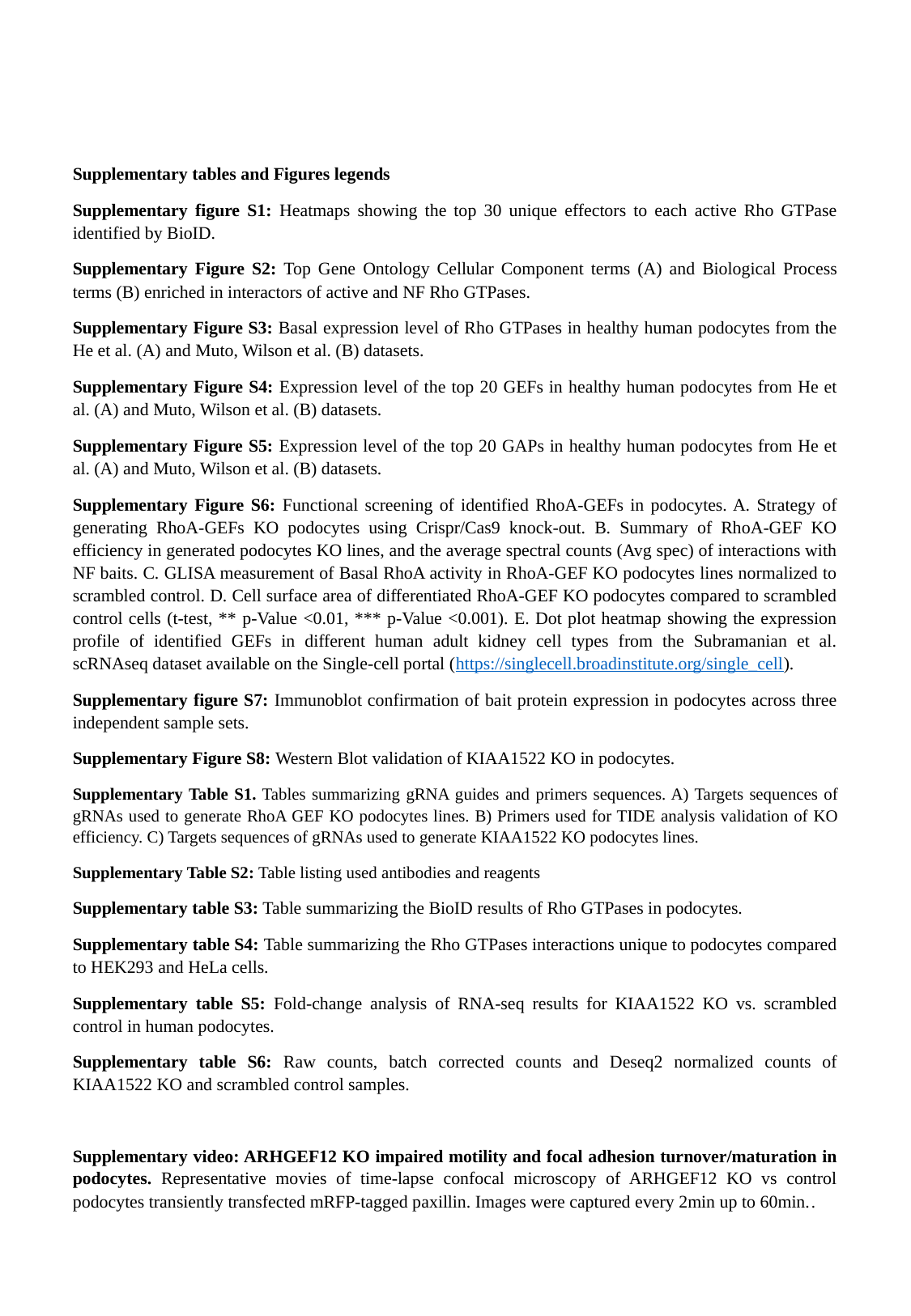

Supplementary tables and Figures legends
Supplementary figure S1: Heatmaps showing the top 30 unique effectors to each active Rho GTPase identified by BioID.
Supplementary Figure S2: Top Gene Ontology Cellular Component terms (A) and Biological Process terms (B) enriched in interactors of active and NF Rho GTPases.
Supplementary Figure S3: Basal expression level of Rho GTPases in healthy human podocytes from the He et al. (A) and Muto, Wilson et al. (B) datasets.
Supplementary Figure S4: Expression level of the top 20 GEFs in healthy human podocytes from He et al. (A) and Muto, Wilson et al. (B) datasets.
Supplementary Figure S5: Expression level of the top 20 GAPs in healthy human podocytes from He et al. (A) and Muto, Wilson et al. (B) datasets.
Supplementary Figure S6: Functional screening of identified RhoA-GEFs in podocytes. A. Strategy of generating RhoA-GEFs KO podocytes using Crispr/Cas9 knock-out. B. Summary of RhoA-GEF KO efficiency in generated podocytes KO lines, and the average spectral counts (Avg spec) of interactions with NF baits. C. GLISA measurement of Basal RhoA activity in RhoA-GEF KO podocytes lines normalized to scrambled control. D. Cell surface area of differentiated RhoA-GEF KO podocytes compared to scrambled control cells (t-test, ** p-Value <0.01, *** p-Value <0.001). E. Dot plot heatmap showing the expression profile of identified GEFs in different human adult kidney cell types from the Subramanian et al. scRNAseq dataset available on the Single-cell portal (https://singlecell.broadinstitute.org/single_cell).
Supplementary figure S7: Immunoblot confirmation of bait protein expression in podocytes across three independent sample sets.
Supplementary Figure S8: Western Blot validation of KIAA1522 KO in podocytes.
Supplementary Table S1. Tables summarizing gRNA guides and primers sequences. A) Targets sequences of gRNAs used to generate RhoA GEF KO podocytes lines. B) Primers used for TIDE analysis validation of KO efficiency. C) Targets sequences of gRNAs used to generate KIAA1522 KO podocytes lines.
Supplementary Table S2: Table listing used antibodies and reagents
Supplementary table S3: Table summarizing the BioID results of Rho GTPases in podocytes.
Supplementary table S4: Table summarizing the Rho GTPases interactions unique to podocytes compared to HEK293 and HeLa cells.
Supplementary table S5: Fold-change analysis of RNA-seq results for KIAA1522 KO vs. scrambled control in human podocytes.
Supplementary table S6: Raw counts, batch corrected counts and Deseq2 normalized counts of KIAA1522 KO and scrambled control samples.
Supplementary video: ARHGEF12 KO impaired motility and focal adhesion turnover/maturation in podocytes. Representative movies of time-lapse confocal microscopy of ARHGEF12 KO vs control podocytes transiently transfected mRFP-tagged paxillin. Images were captured every 2min up to 60min..

## Slide 2
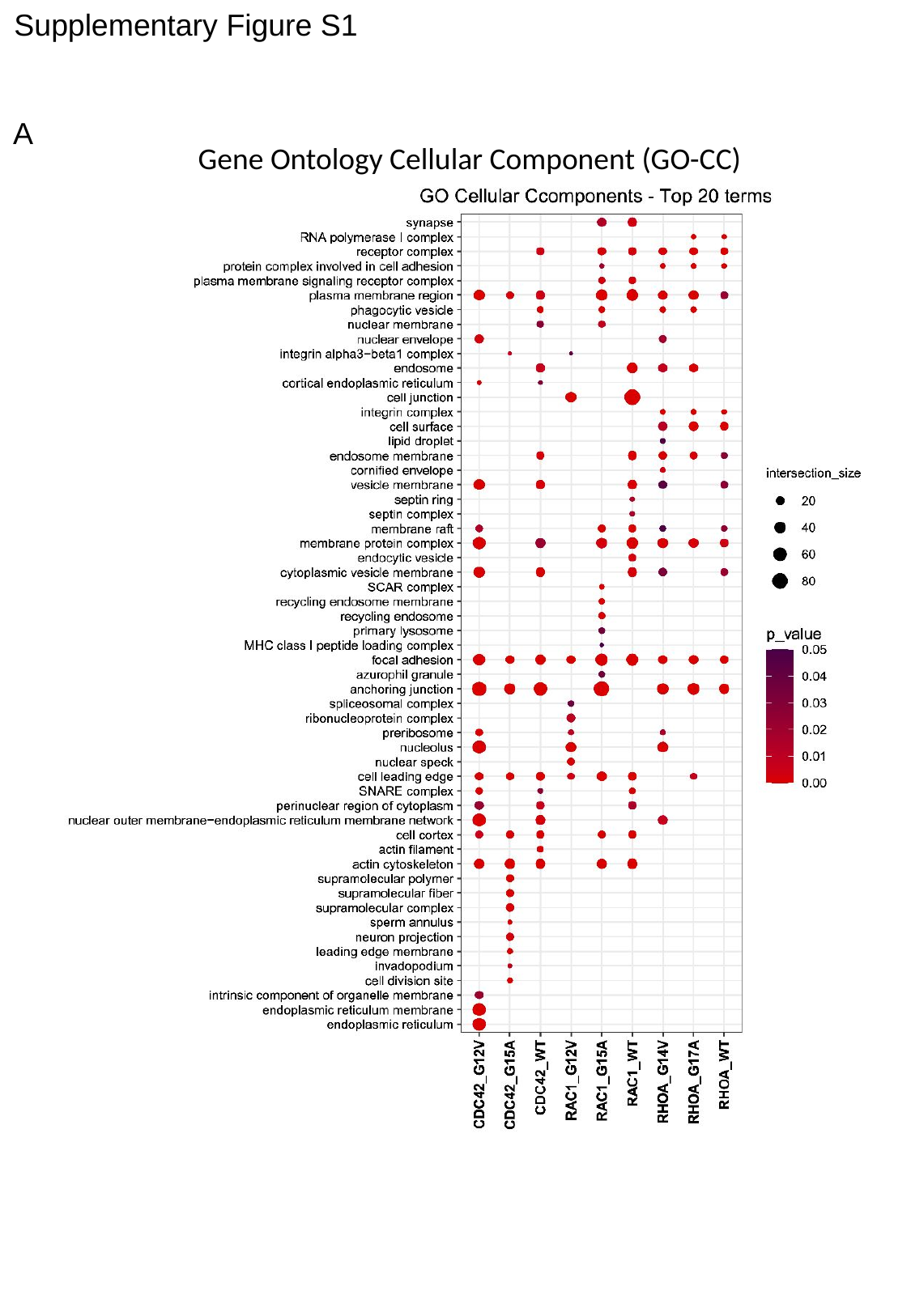

Supplementary Figure S1
A
Gene Ontology Cellular Component (GO-CC)

## Slide 3
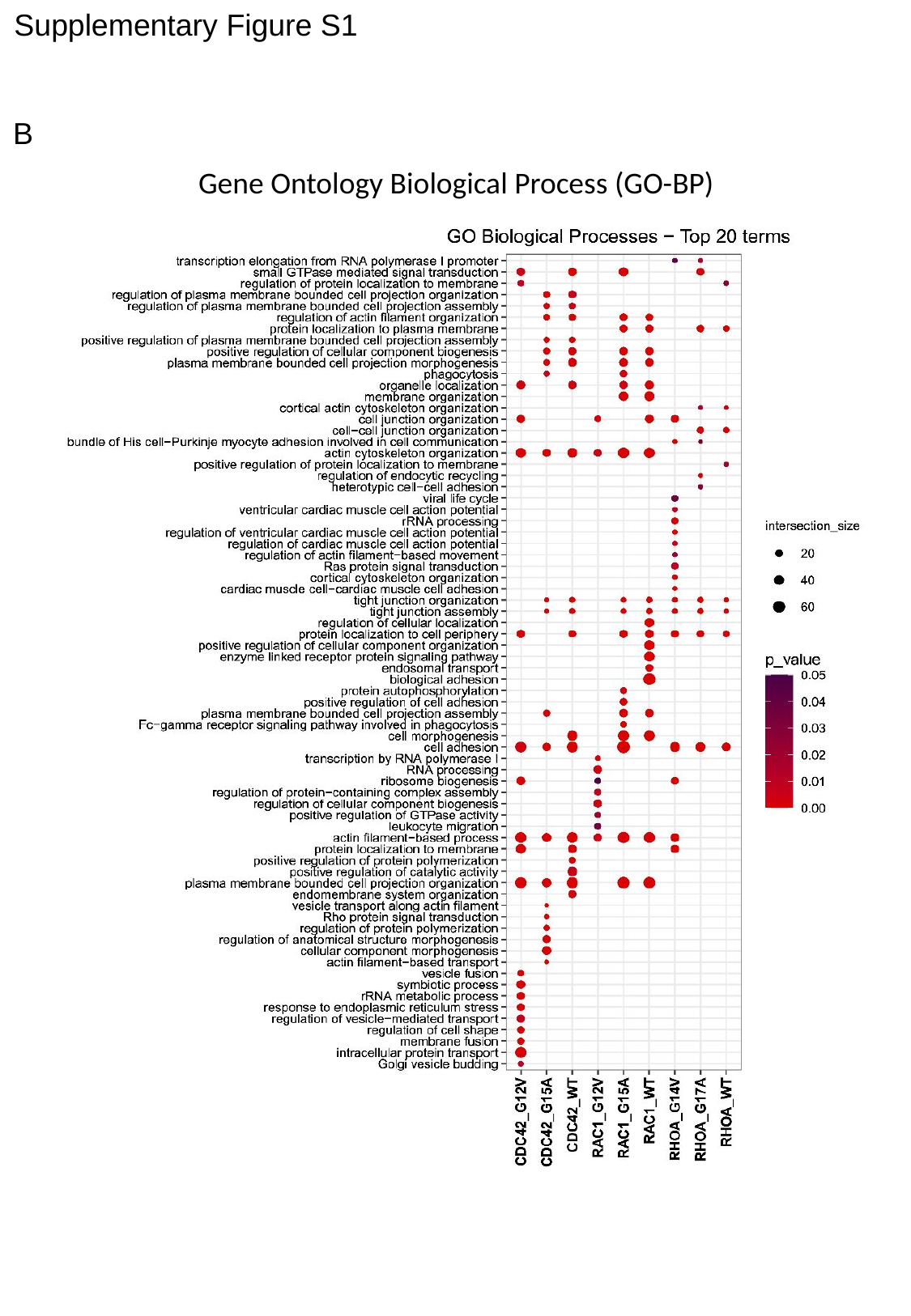

Supplementary Figure S1
B
Gene Ontology Biological Process (GO-BP)

## Slide 4
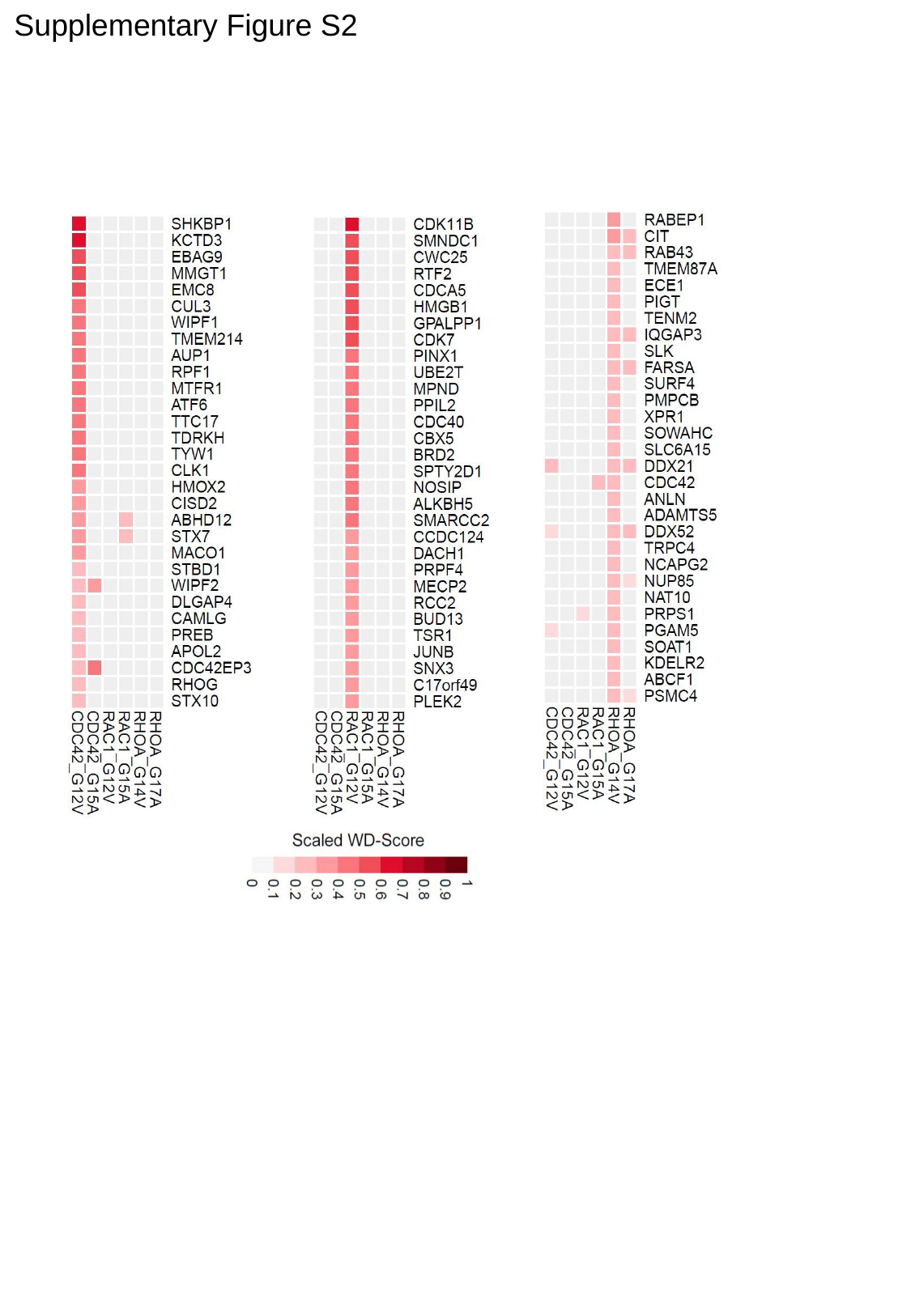

Supplementary Figure S2

## Slide 5
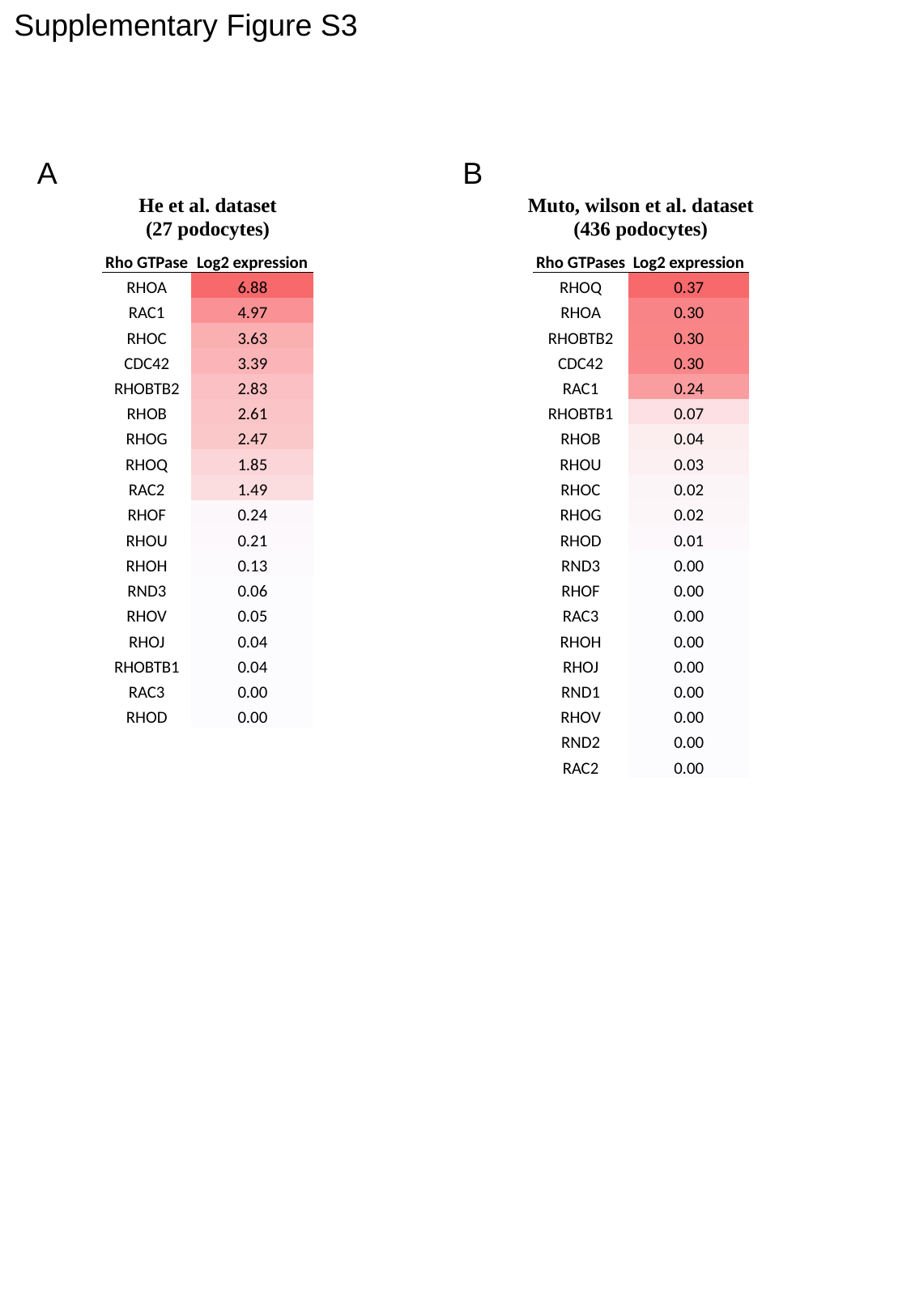

Supplementary Figure S3
A
B
He et al. dataset
(27 podocytes)
Muto, wilson et al. dataset
(436 podocytes)
| Rho GTPase | Log2 expression |
| --- | --- |
| RHOA | 6.88 |
| RAC1 | 4.97 |
| RHOC | 3.63 |
| CDC42 | 3.39 |
| RHOBTB2 | 2.83 |
| RHOB | 2.61 |
| RHOG | 2.47 |
| RHOQ | 1.85 |
| RAC2 | 1.49 |
| RHOF | 0.24 |
| RHOU | 0.21 |
| RHOH | 0.13 |
| RND3 | 0.06 |
| RHOV | 0.05 |
| RHOJ | 0.04 |
| RHOBTB1 | 0.04 |
| RAC3 | 0.00 |
| RHOD | 0.00 |
| Rho GTPases | Log2 expression |
| --- | --- |
| RHOQ | 0.37 |
| RHOA | 0.30 |
| RHOBTB2 | 0.30 |
| CDC42 | 0.30 |
| RAC1 | 0.24 |
| RHOBTB1 | 0.07 |
| RHOB | 0.04 |
| RHOU | 0.03 |
| RHOC | 0.02 |
| RHOG | 0.02 |
| RHOD | 0.01 |
| RND3 | 0.00 |
| RHOF | 0.00 |
| RAC3 | 0.00 |
| RHOH | 0.00 |
| RHOJ | 0.00 |
| RND1 | 0.00 |
| RHOV | 0.00 |
| RND2 | 0.00 |
| RAC2 | 0.00 |

## Slide 6
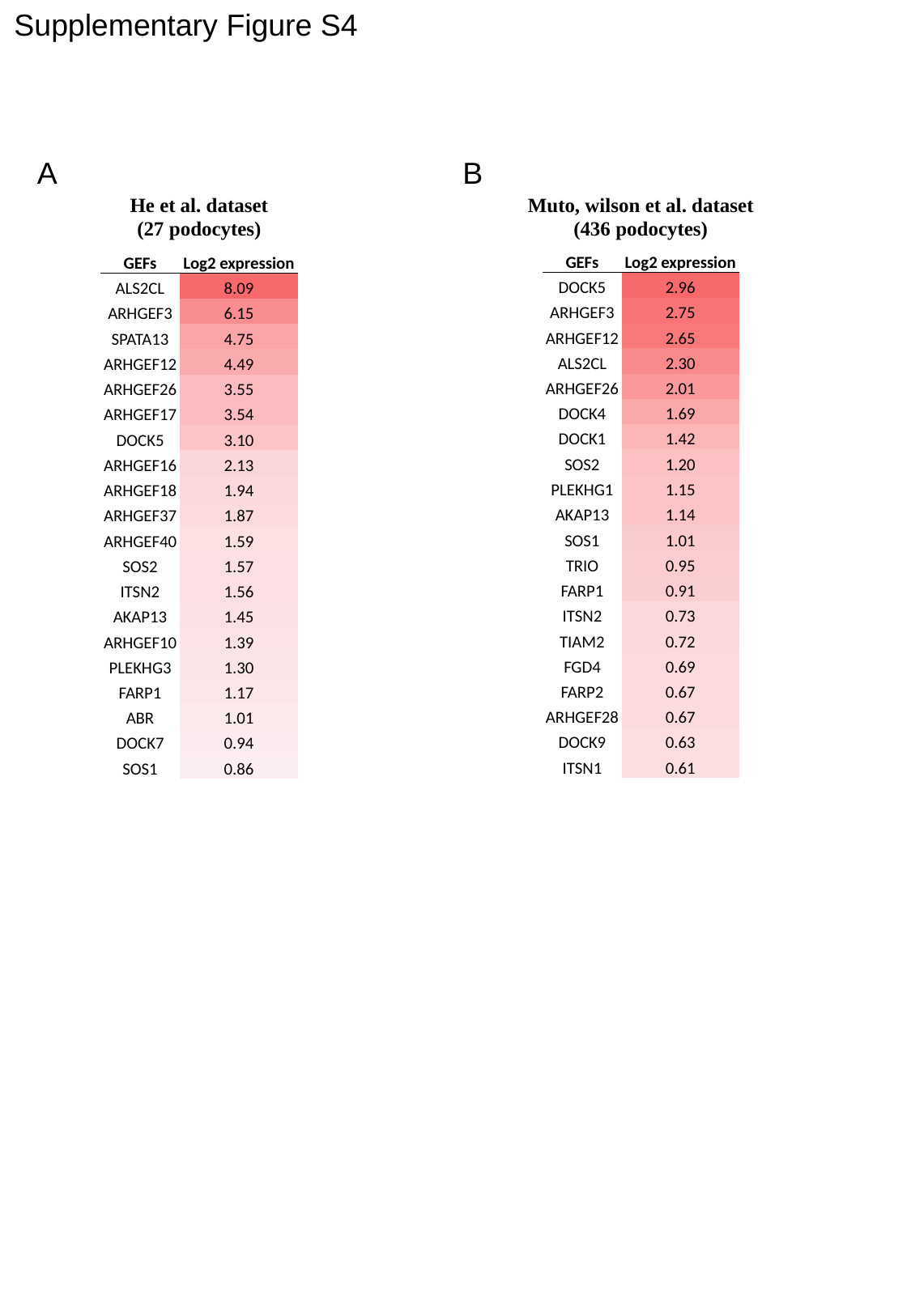

Supplementary Figure S4
A
B
He et al. dataset
(27 podocytes)
Muto, wilson et al. dataset
(436 podocytes)
| GEFs | Log2 expression |
| --- | --- |
| DOCK5 | 2.96 |
| ARHGEF3 | 2.75 |
| ARHGEF12 | 2.65 |
| ALS2CL | 2.30 |
| ARHGEF26 | 2.01 |
| DOCK4 | 1.69 |
| DOCK1 | 1.42 |
| SOS2 | 1.20 |
| PLEKHG1 | 1.15 |
| AKAP13 | 1.14 |
| SOS1 | 1.01 |
| TRIO | 0.95 |
| FARP1 | 0.91 |
| ITSN2 | 0.73 |
| TIAM2 | 0.72 |
| FGD4 | 0.69 |
| FARP2 | 0.67 |
| ARHGEF28 | 0.67 |
| DOCK9 | 0.63 |
| ITSN1 | 0.61 |
| GEFs | Log2 expression |
| --- | --- |
| ALS2CL | 8.09 |
| ARHGEF3 | 6.15 |
| SPATA13 | 4.75 |
| ARHGEF12 | 4.49 |
| ARHGEF26 | 3.55 |
| ARHGEF17 | 3.54 |
| DOCK5 | 3.10 |
| ARHGEF16 | 2.13 |
| ARHGEF18 | 1.94 |
| ARHGEF37 | 1.87 |
| ARHGEF40 | 1.59 |
| SOS2 | 1.57 |
| ITSN2 | 1.56 |
| AKAP13 | 1.45 |
| ARHGEF10 | 1.39 |
| PLEKHG3 | 1.30 |
| FARP1 | 1.17 |
| ABR | 1.01 |
| DOCK7 | 0.94 |
| SOS1 | 0.86 |

## Slide 7
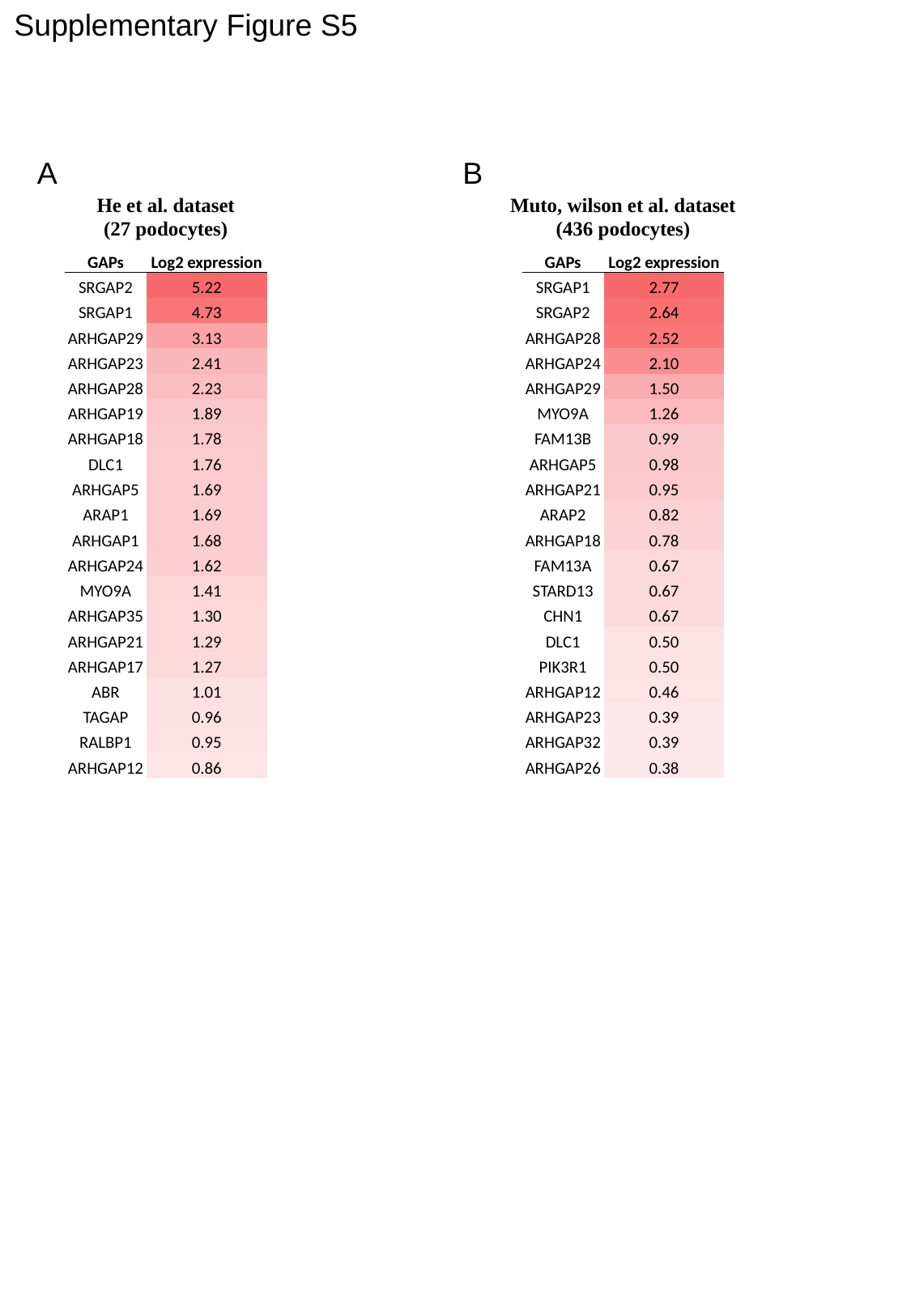

Supplementary Figure S5
A
B
He et al. dataset
(27 podocytes)
Muto, wilson et al. dataset
(436 podocytes)
| GAPs | Log2 expression |
| --- | --- |
| SRGAP2 | 5.22 |
| SRGAP1 | 4.73 |
| ARHGAP29 | 3.13 |
| ARHGAP23 | 2.41 |
| ARHGAP28 | 2.23 |
| ARHGAP19 | 1.89 |
| ARHGAP18 | 1.78 |
| DLC1 | 1.76 |
| ARHGAP5 | 1.69 |
| ARAP1 | 1.69 |
| ARHGAP1 | 1.68 |
| ARHGAP24 | 1.62 |
| MYO9A | 1.41 |
| ARHGAP35 | 1.30 |
| ARHGAP21 | 1.29 |
| ARHGAP17 | 1.27 |
| ABR | 1.01 |
| TAGAP | 0.96 |
| RALBP1 | 0.95 |
| ARHGAP12 | 0.86 |
| GAPs | Log2 expression |
| --- | --- |
| SRGAP1 | 2.77 |
| SRGAP2 | 2.64 |
| ARHGAP28 | 2.52 |
| ARHGAP24 | 2.10 |
| ARHGAP29 | 1.50 |
| MYO9A | 1.26 |
| FAM13B | 0.99 |
| ARHGAP5 | 0.98 |
| ARHGAP21 | 0.95 |
| ARAP2 | 0.82 |
| ARHGAP18 | 0.78 |
| FAM13A | 0.67 |
| STARD13 | 0.67 |
| CHN1 | 0.67 |
| DLC1 | 0.50 |
| PIK3R1 | 0.50 |
| ARHGAP12 | 0.46 |
| ARHGAP23 | 0.39 |
| ARHGAP32 | 0.39 |
| ARHGAP26 | 0.38 |

## Slide 8
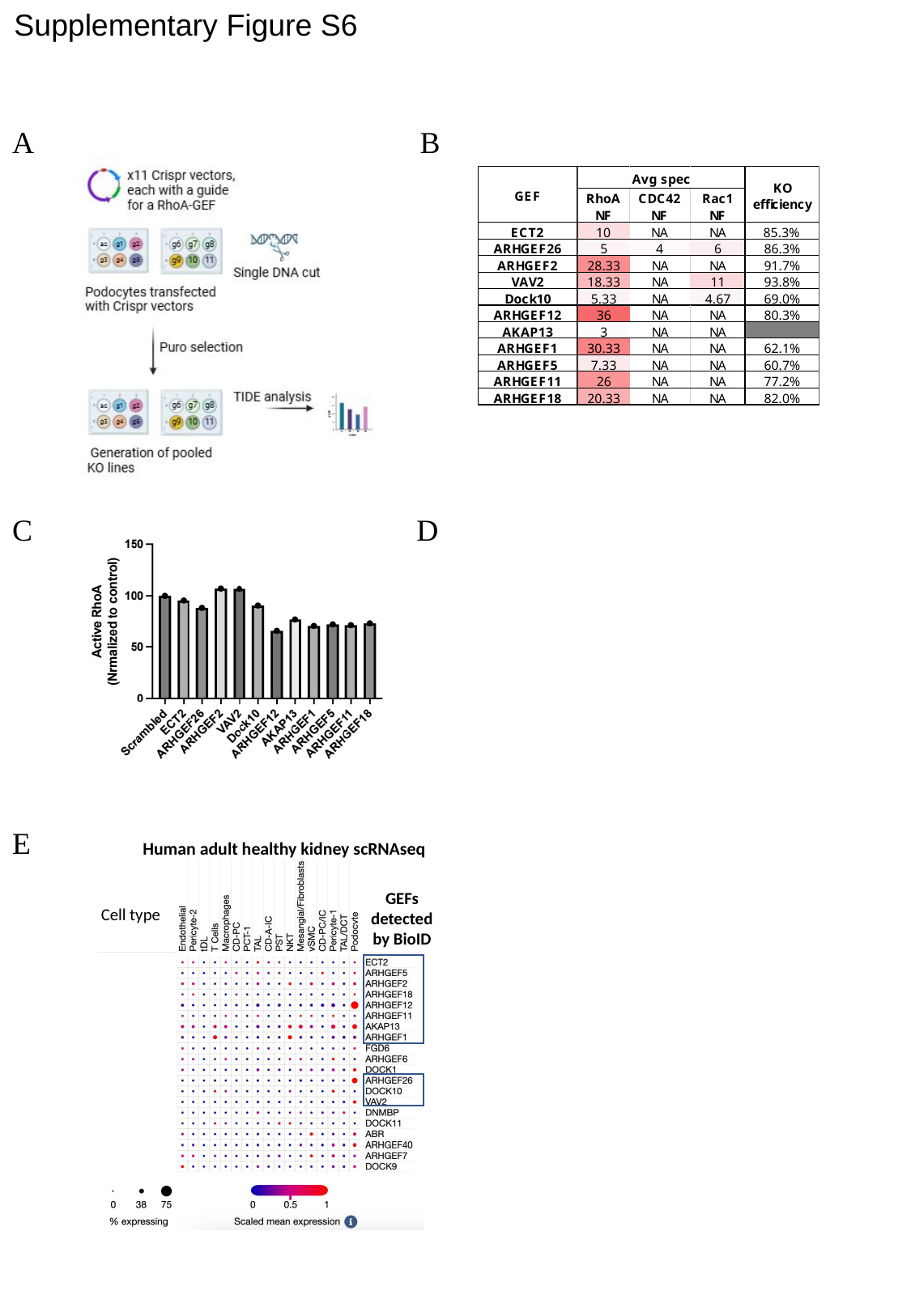

Supplementary Figure S6
A
B
C
D
E
Human adult healthy kidney scRNAseq
GEFs detected by BioID
Cell type

## Slide 9
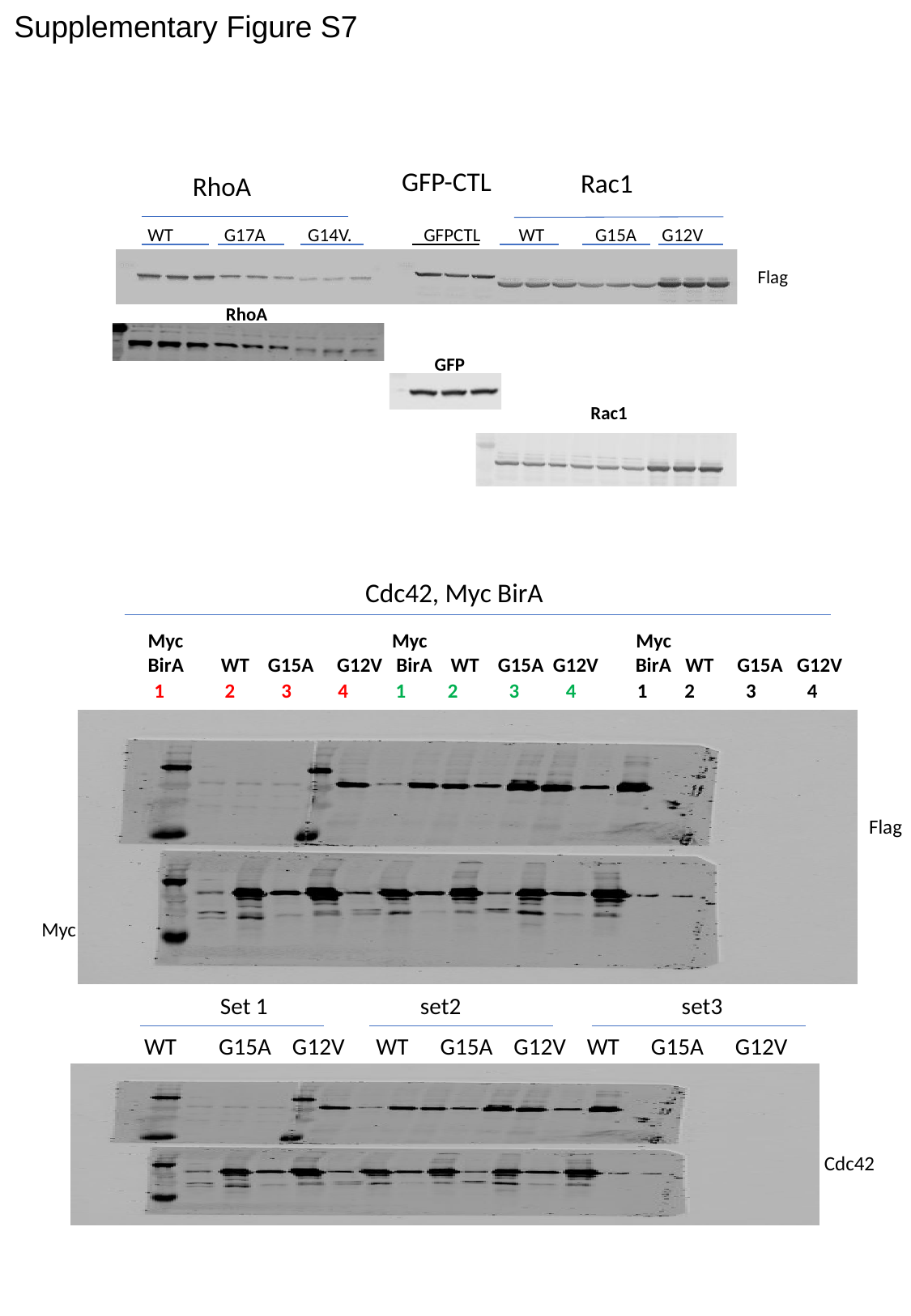

Supplementary Figure S7
GFP-CTL
Rac1
RhoA
WT G17A G14V. GFPCTL WT G15A G12V
Flag
RhoA
GFP
Rac1
Cdc42, Myc BirA
Myc Myc Myc
BirA WT G15A G12V BirA WT G15A G12V BirA WT G15A G12V
 1 2 3 4 1 2 3 4 1 2 3 4
Flag
Myc
Set 1 set2 set3
WT G15A G12V WT G15A G12V WT G15A G12V
Cdc42

## Slide 10
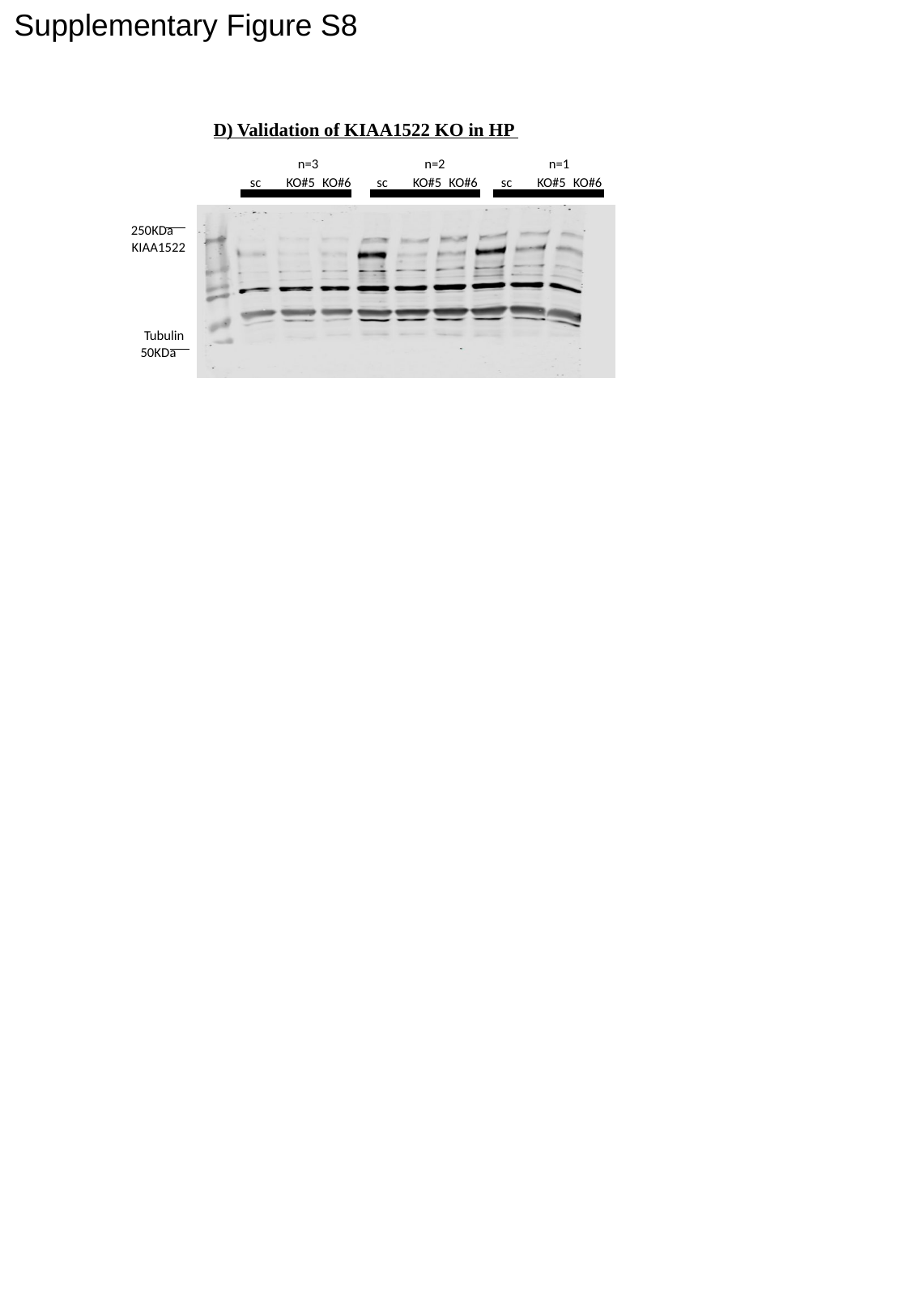

Supplementary Figure S8
D) Validation of KIAA1522 KO in HP
n=2
n=1
n=3
KO#5
KO#6
KO#5
KO#6
sc
sc
KO#5
KO#6
sc
250KDa
KIAA1522
Tubulin
50KDa

## Slide 11
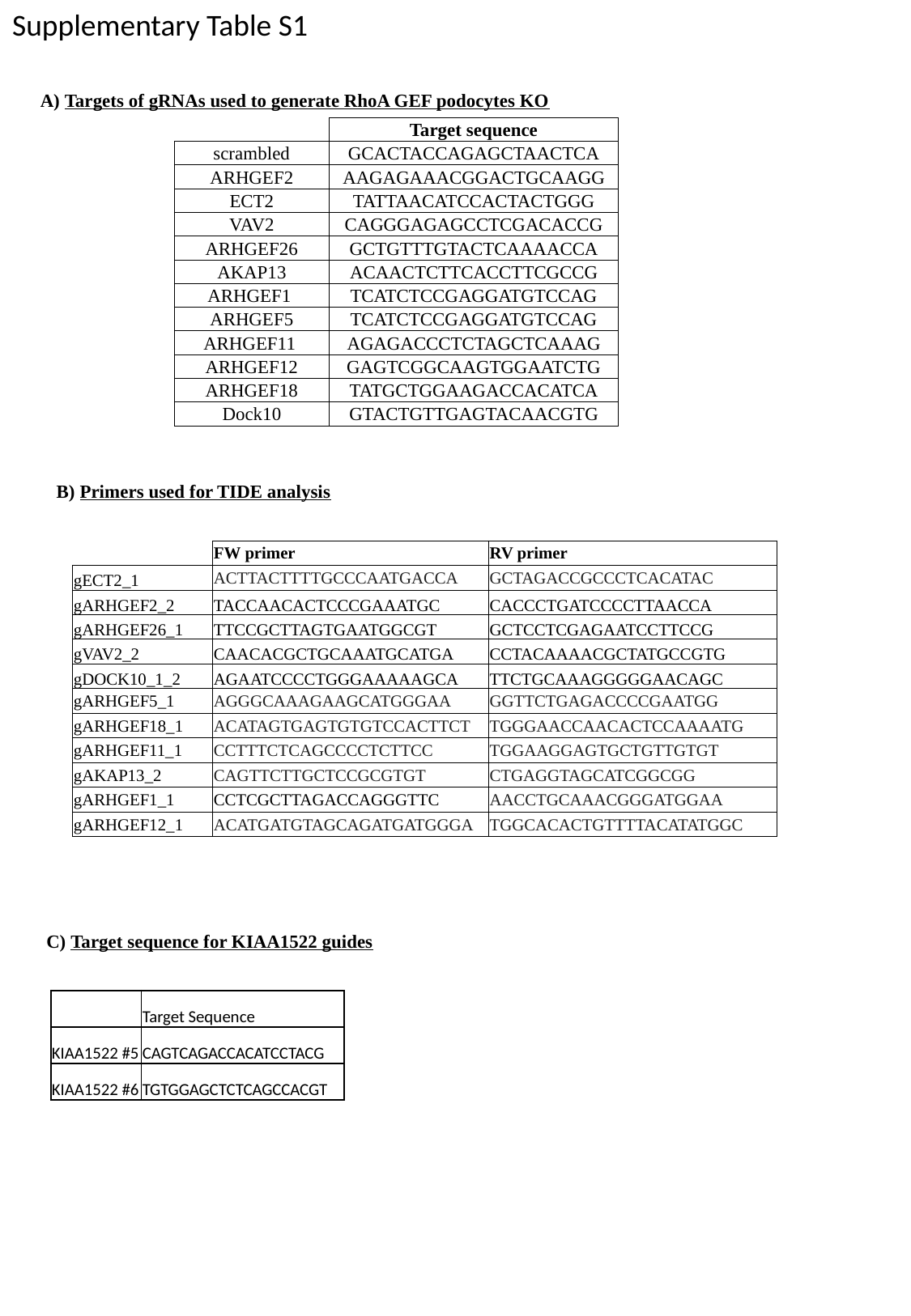

Supplementary Table S1
A) Targets of gRNAs used to generate RhoA GEF podocytes KO
| | Target sequence |
| --- | --- |
| scrambled | GCACTACCAGAGCTAACTCA |
| ARHGEF2 | AAGAGAAACGGACTGCAAGG |
| ECT2 | TATTAACATCCACTACTGGG |
| VAV2 | CAGGGAGAGCCTCGACACCG |
| ARHGEF26 | GCTGTTTGTACTCAAAACCA |
| AKAP13 | ACAACTCTTCACCTTCGCCG |
| ARHGEF1 | TCATCTCCGAGGATGTCCAG |
| ARHGEF5 | TCATCTCCGAGGATGTCCAG |
| ARHGEF11 | AGAGACCCTCTAGCTCAAAG |
| ARHGEF12 | GAGTCGGCAAGTGGAATCTG |
| ARHGEF18 | TATGCTGGAAGACCACATCA |
| Dock10 | GTACTGTTGAGTACAACGTG |
B) Primers used for TIDE analysis
| | FW primer | RV primer |
| --- | --- | --- |
| gECT2\_1 | ACTTACTTTTGCCCAATGACCA | GCTAGACCGCCCTCACATAC |
| gARHGEF2\_2 | TACCAACACTCCCGAAATGC | CACCCTGATCCCCTTAACCA |
| gARHGEF26\_1 | TTCCGCTTAGTGAATGGCGT | GCTCCTCGAGAATCCTTCCG |
| gVAV2\_2 | CAACACGCTGCAAATGCATGA | CCTACAAAACGCTATGCCGTG |
| gDOCK10\_1\_2 | AGAATCCCCTGGGAAAAAGCA | TTCTGCAAAGGGGGAACAGC |
| gARHGEF5\_1 | AGGGCAAAGAAGCATGGGAA | GGTTCTGAGACCCCGAATGG |
| gARHGEF18\_1 | ACATAGTGAGTGTGTCCACTTCT | TGGGAACCAACACTCCAAAATG |
| gARHGEF11\_1 | CCTTTCTCAGCCCCTCTTCC | TGGAAGGAGTGCTGTTGTGT |
| gAKAP13\_2 | CAGTTCTTGCTCCGCGTGT | CTGAGGTAGCATCGGCGG |
| gARHGEF1\_1 | CCTCGCTTAGACCAGGGTTC | AACCTGCAAACGGGATGGAA |
| gARHGEF12\_1 | ACATGATGTAGCAGATGATGGGA | TGGCACACTGTTTTACATATGGC |
C) Target sequence for KIAA1522 guides
| | Target Sequence |
| --- | --- |
| KIAA1522 #5 | CAGTCAGACCACATCCTACG |
| KIAA1522 #6 | TGTGGAGCTCTCAGCCACGT |
Future studies are needed to reveal KIAA1522 functions

## Slide 12
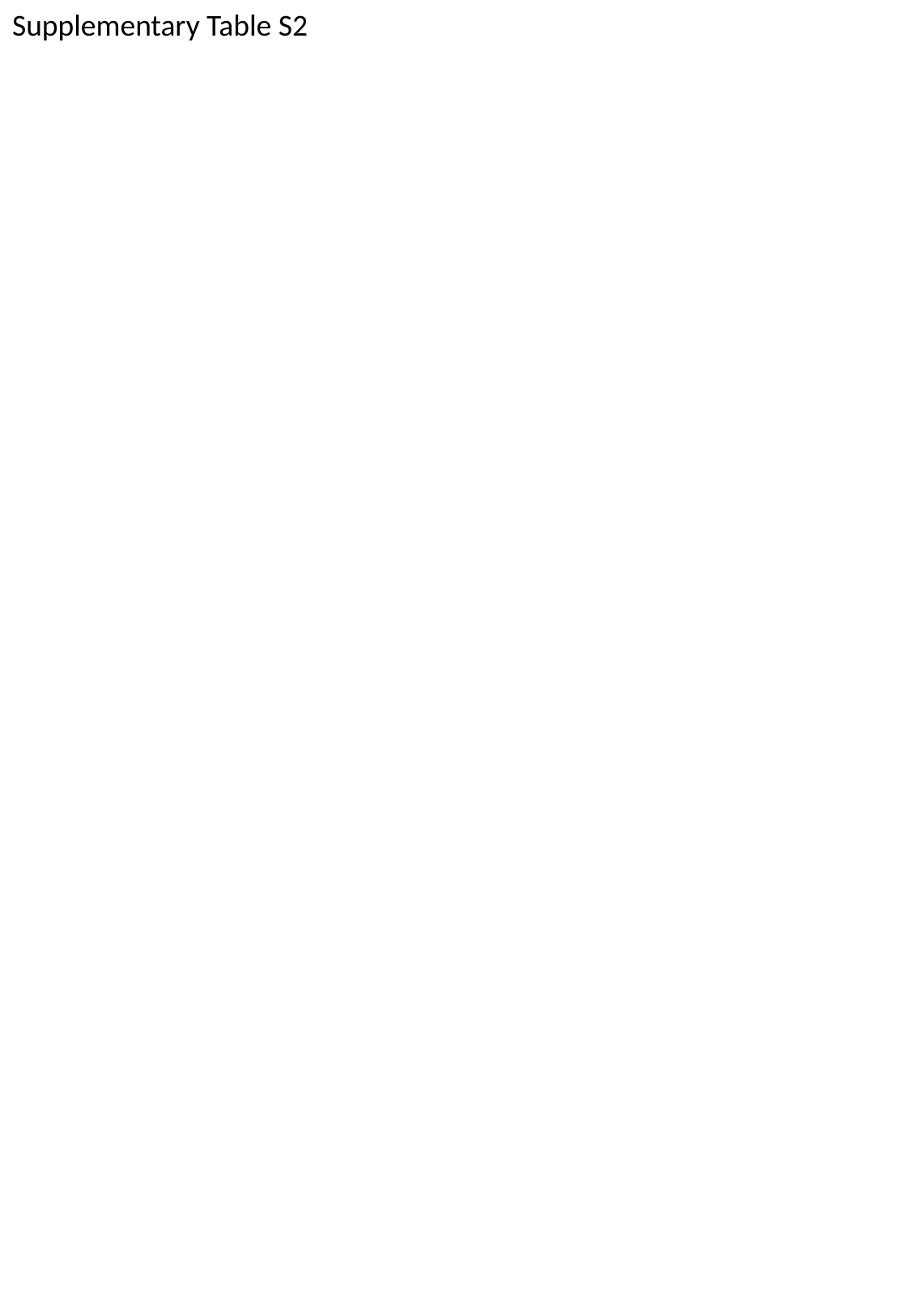

Supplementary Table S2
